# Coronin 2B deficiency induces nucleolar stress and neuronal apoptosis

**DOI:** 10.1038/s41419-024-06852-x

**Published:** 2024-06-27

**Authors:** Hongjiao Wu, Yujie Yang, Wanying Yi, Yue Qiu, Shuangshuang Ma, Jinying Xu, Yingying Fan, Yuewen Chen, Yu Chen

**Affiliations:** 1 Chinese Academy of Sciences Key Laboratory of Brain Connectome and Manipulation, Shenzhen Key Laboratory of Translational Research for Brain Diseases, The Brain Cognition and Brain Disease Institute, Shenzhen Institute of Advanced Technology, Chinese Academy of Sciences, ; Shenzhen–Hong Kong Institute of Brain Science—Shenzhen Fundamental Research Institutions, Shenzhen, Guangdong 518055 China; 2 SIAT-HKUST Joint Laboratory for Brain Science, Chinese Academy of Sciences, Shenzhen, China; 3 University of the Chinese Academy of Sciences, Beijing, 100049 China; 4 Guangdong Provincial Key Laboratory of Brain Science, Disease and Drug Development, HKUST Shenzhen Research Institute, Shenzhen, Guangdong 518057 China

**Keywords:** Cell death in the nervous system, Senescence

## Abstract

In eukaryotes, the nucleolus is the critical non-membranous organelle within nuclei that is responsible for ribosomal DNA (rDNA) transcription and ribosome biogenesis. The transcription of rDNA, a rate-limiting step for ribosome biogenesis, is tightly regulated to meet the demand for global protein synthesis in response to cell physiology, especially in neurons, which undergo rapid changes in morphology and protein composition during development and synaptic plasticity. However, it is unknown how the pre-initiation complex for rDNA transcription is efficiently assembled within the nucleolus in neurons. Here, we report that the nucleolar protein, coronin 2B, regulates rDNA transcription and maintains nucleolar function through direct interaction with upstream binding factor (UBF), an activator of RNA polymerase I transcriptional machinery. We show that coronin 2B knockdown impairs the formation of the transcription initiation complex, inhibits rDNA transcription, destroys nucleolar integrity, and ultimately induces nucleolar stress. In turn, coronin 2B-mediated nucleolar stress leads to p53 stabilization and activation, eventually resulting in neuronal apoptosis. Thus, we identified that coronin 2B coordinates with UBF to regulate rDNA transcription and maintain proper nucleolar function in neurons.

## Introduction

Although neurons are postmitotic cells with limited growth potential, their metabolic demands are extremely high owing to the maintenance of the neuronal network and thousands of synaptic contacts. Therefore, neurons require greater ribosome production than other cell types [1, 2]. In eukaryotic cells, the nucleolus is the site of ribosome biogenesis, which is a series of sequential processes including ribosomal DNA (rDNA) transcription, pre-ribosomal RNA processing, and ribosomal subunit assembly [3, 4]. The transcription of rDNA is the tightly regulated, rate-limiting step of ribosome biogenesis [5]. Efficient rDNA transcription begins with the formation of the transcription pre-initiation complex, which comprises selective factor 1 (SL-1), upstream binding factor (UBF), and RNA polymerase I (Pol I). A crucial step of pre-initiation complex assembly is the binding of UBF to the upstream and core promoter elements of the rDNA promoter. This is followed by the recruitment of SL-1 to form the UBF/SL-1 complex, which facilitates the recruitment of Pol I transcriptional machinery to rDNA. The functional rDNA transcription initiation complex is subsequently assembled at the rDNA promoter [4, 6].

Technical advances in cryo-electron microscopy have resolved the structures of Pol I, providing important insights into the molecular architecture of the Pol I transcriptional machinery, including the molecular model for basal Pol I transcription initiation [7], the Pol I initiation mechanisms at the early initiation stage [8], and the relationship between polymerase conformations and activity states [9]. These recent structural investigations have greatly advanced our understanding of the transcriptional machinery of Pol I. Accordingly, the identification of novel components or regulators of Pol I transcription initiation may further advance our understanding of the regulation of ribosome biogenesis in the nervous system.

Given that the repetitive structure and high transcription rate of rDNA make the nucleolus vulnerable to various insults [10], the nucleolus is proposed to be a critical stress sensor[11, 12]. Accordingly, nucleolar stress, defined as the inhibition of ribosome biogenesis or destruction of nucleolar integrity or function, ultimately leads to the disruption of cellular homeostasis [13, 14]. Several studies indicate that the p53-dependent signaling pathway is critical for sensing nucleolar stress [15]. In response to nucleolar stress, several ribosomal proteins translocate from the nucleolus into the nucleoplasm, where they bind to mouse double minute 2 homolog (MDM2), preventing p53 from binding to MDM2 and hence its MDM2-mediated ubiquitination and degradation. In turn, the stabilization and accumulation of p53 ultimately initiate p53-dependent cell cycle arrest, senescence, and apoptosis [15]. In addition, a hallmark of nucleolar stress, the nucleoplasmic translocation of nucleophosmin (NPM1 or B23) following S-glutathionylation is a prerequisite for the stress-induced activation of p53 regardless of the presence of ribosomal proteins [16].

Owing to the particularly great demands of ribosome biogenesis in neurons, altered nucleolar functioning is closely associated with neuronal homeostasis [1]. In mice, genetic ablation of TIF-IA, a transcription factor that regulates the activity of Pol I, impairs rDNA transcription and disrupts nucleolar structure, ultimately leading to neuronal apoptosis and chronic neurodegeneration [17, 18]. A nucleolus-specific long noncoding RNA, LoNA, negatively regulates rDNA transcription and ribosome biogenesis in the nucleolus, ultimately negatively regulating protein synthesis. Knockdown of LoNA promotes ribosome transport to synapses, resulting in elevated levels of AMPA and NMDA receptors, enhanced synaptic plasticity, long-term potentiation, and memory consolidation [19]. These studies collectively indicate that inhibition of rDNA transcription is a major cause of nucleolar dysfunction in the nervous system. However, despite the intriguing role of the nucleolus as a sensor involved in neural protection, the molecular mechanisms that underlie the fine-tuned regulation of rDNA transcription in neurons remain unclear.

Meanwhile, coronins are a family of evolutionarily conserved, actin-binding proteins that regulate actin dynamics via the actin-polymerizing nucleator, Arp2/3 complex, along with actin-severing ADF/cofilin [20, 21]. We and others have shown that coronin 2B is mainly expressed in the nervous system and regulates neural development [21–23]. In the present study, we observed that coronin 2B is mainly enriched in neuronal nucleoli and colocalizes with UBF, a core protein involved in the assembly of Pol I transcriptional machinery in cultured primary neurons. In addition, we found that coronin 2B knockdown impairs the formation of the transcription initiation complex, thereby inhibiting rDNA transcription, disrupting nucleolar integrity, and inducing nucleolar stress, which ultimately leads to neuronal apoptosis. Thus, our findings suggest that coronin 2B is required for the assembly of the rDNA transcription complex and maintenance of nucleolar function.

## Results

### Coronin 2B is enriched in the nucleoli of neurons

**Fig. 1:**
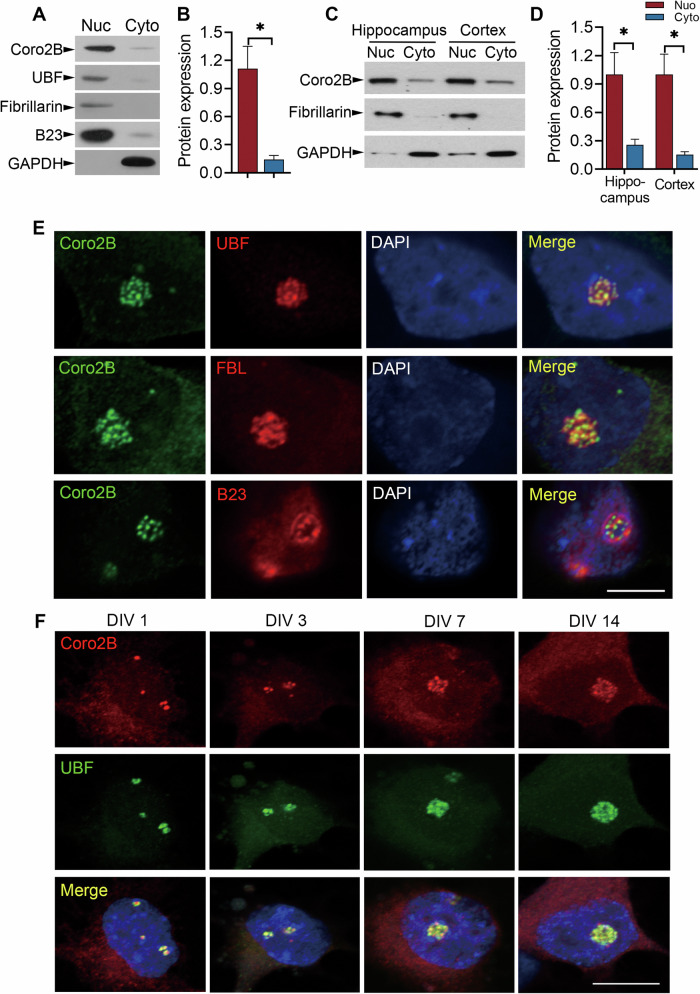
Coronin 2B specifically localizes at the nucleolus in neurons. **A**–**D** Coronin 2B expression is enriched in the nucleus in neurons. Cytoplasmic and nuclear fractions were isolated from cortical neurons cultured for 14 days (**A**, **B**) and rat brain tissues (**C**, **D**). Western blot analysis was performed using antibodies against coronin 2B; the nucleolar markers, UBF, FBL, and B23; and the cytoplasmic marker, GAPDH (**A**, **C**). Coronin 2B was quantified by immunoblotting and normalized to that of GAPDH and FBL (**B**, **D**). Data are mean ± SEM (from three independent experiments, unpaired Student’s *t*-test, *p < 0.05). Nuc*:* nucleolar, Cyto: cytoplasmic. **E** Coronin 2B localizes at the nucleolus. Dissociated hippocampal neurons were cultured for 14 days and then immunostained with anti-coronin 2B (green) and antibodies against the nucleolar markers, UBF, FBL, and B23 (red). Scale bars: 5 µm. **F** Morphological variation during different development stages of the nucleolus. DIV days in vitro. Scale bars: 5 µm.

To further characterize the function of coronin 2B in neurons, we first examined its subcellular localization in cultured cortical neurons. In the nucleocytoplasmic fraction, coronin 2B protein was highly enriched in the nuclei of cultured cortical neurons (Fig. 1A, B) and rat brain tissues (Fig. 1C, D). Subsequent examination of the subcellular localization of coronin 2B by immunostaining showed that coronin 2B is mainly present in the nucleolus and colocalizes with the nucleolar marker, UBF, but not with fibrillarin (FBL) or B23, which are nucleolar proteins required for rRNA processing (Fig. 1E). Importantly, the nucleolar enrichment of coronin 2B increased continuously during the maturation of cultured neurons from 1 to 14 days in vitro (Fig. 1F). In addition, we confirmed the specificity of coronin 2B signals in neuronal nucleoli upon neutralization by administration of anti-coronin 2B antibody with the corresponding blocking peptide (Supplementary Fig. 1).

**Fig. 2:**
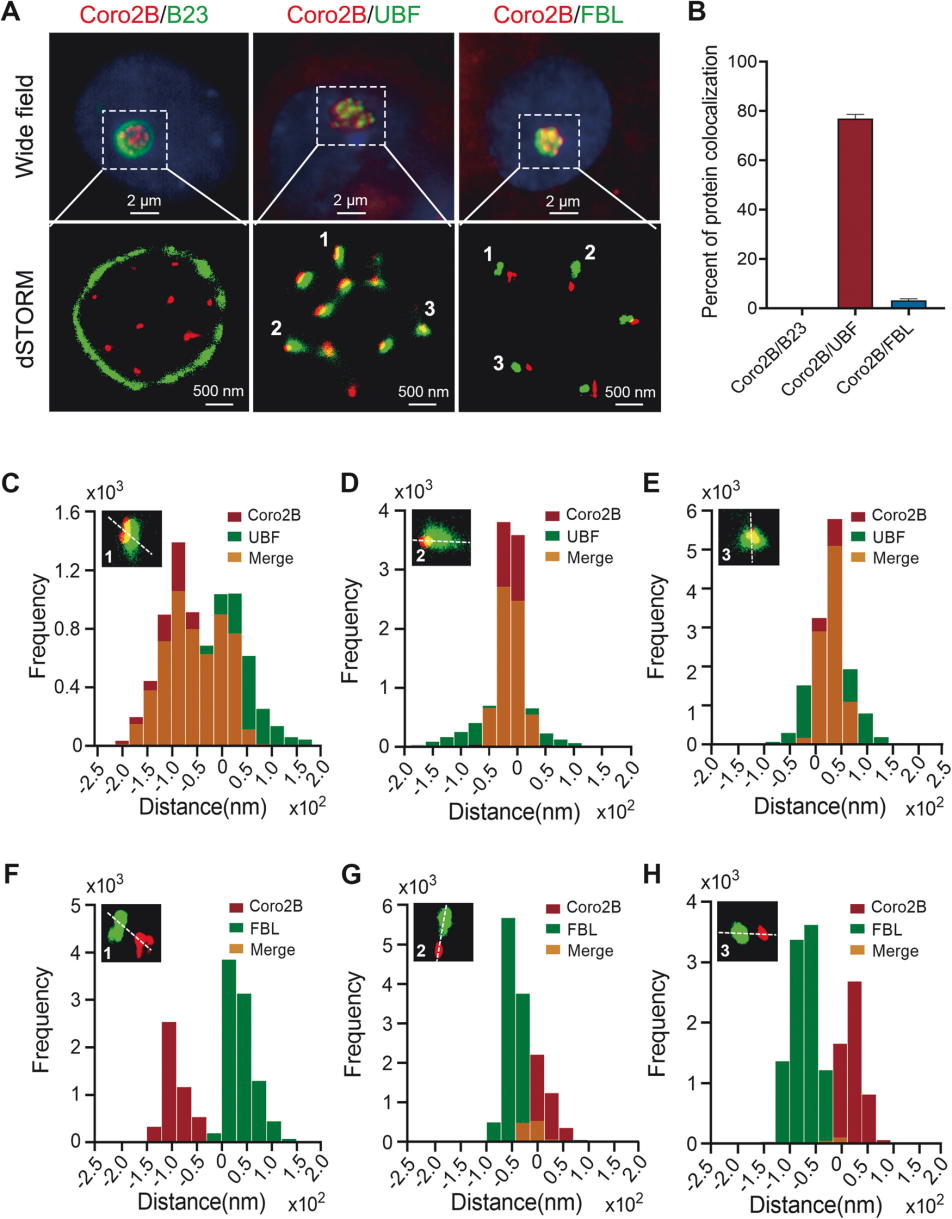
Coronin 2B colocalizes with UBF in neuronal nucleoli. **A**, **B** Dissociated hippocampal neurons were cultured for 14 days and then immunostained with the indicated antibodies. Images of coronin 2B (red) and the nucleolar markers B23, UBF, and FBL (green) were acquired with wide-field (top) and two-color direct stochastic optical reconstruction microscopy (dSTORM, bottom) using Nanoimager (**A**). Protein co-localization was quantified by ImarisColoc (**B**). **C**–**H** The spatial distributions of regions of interest for localization were determined by line scan quantification.

We then validated the co-localization of coronin 2B with nucleolar markers by performing direct stochastic optical reconstruction microscopy (dSTORM) (Fig. 2A). Accordingly, coronin 2B exclusively co-localized with UBF. Approximately 76% of UBF clusters co-localized with coronin 2B signals, whereas less than 5% co-localized with nucleolar marker FBL and almost none with B23 (Fig. 2B). Quantification of the frequencies of localization and distance further indicated that most coronin 2B co-localized with UBF (Fig. 2C–E) followed by FBL (Fig. 2F–H). These results collectively suggest that coronin 2B is a nucleolar component that is predominantly enriched in UBF-containing regions.

### Coronin 2B regulates the formation of the rDNA transcription initiation complex

**Fig. 3:**
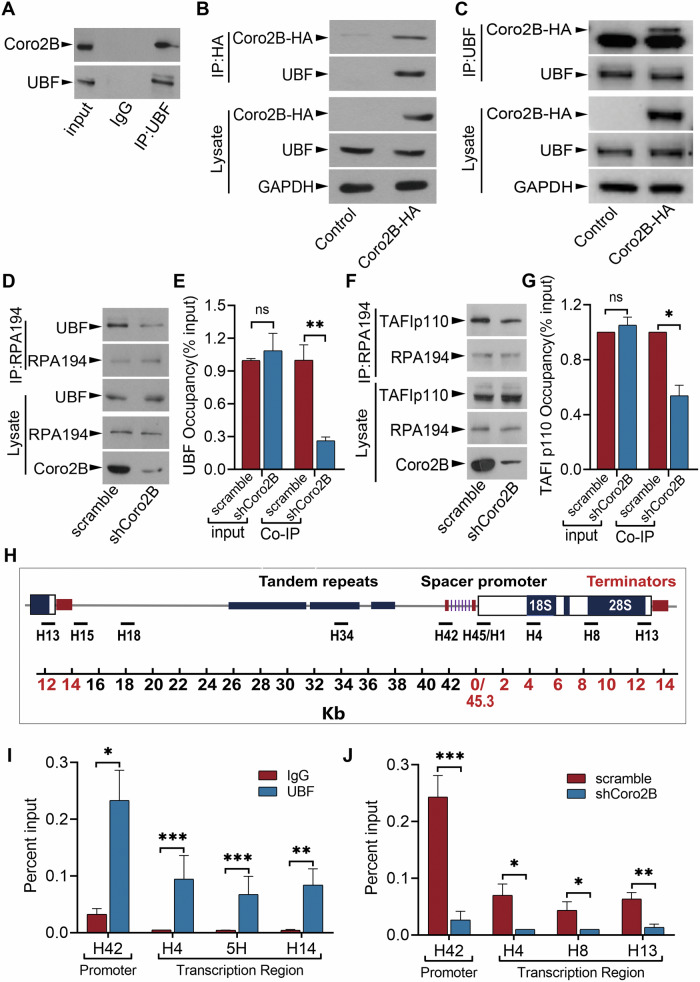
Coronin 2B knockdown impairs the formation of the transcription initiation complex. **A** Coronin 2B is associated with UBF in vivo. UBF was immunoprecipitated from the nuclear extracts of cortical neurons cultured for 14 days. Western blot analysis was performed to determine the associations of coronin 2B and UBF with their respective antibodies and mouse IgG as a negative control. **B**, **C** Coronin 2B interacts with UBF in vitro. Cell lysates from HEK293T cells expressing coronin 2B-HA or pcDNA3.0 vector control were subjected to immunoprecipitation with antibody against HA (**B**) and UBF (**C**), respectively. Western blot analysis revealed the reciprocal interaction between UBF and coronin 2B. **D**–**G** The formation of the transcription initiation complex is impaired in coronin 2B-deficient cortical neurons. Cortical neurons were cultured for 9 days and then infected with shCoro2B lentivirus or corresponding scramble control. After 5 days, Pol I was immunoprecipitated from the nuclear extracts with an antibody against RPA194. The co-immunoprecipitation of UBF and TAFIp110 was analyzed on immunoblots with respective antibodies. The quantification is shown in (**E**) and (**G**) (from three independent experiments, unpaired Student’s *t*-test; *p < 0.05, **p < 0.01, ***p < 0.001, ns not significant). **H** Schematic of rDNA repeats and primers used for chromatin immunoprecipitation (ChIP) assay. H42 is at the promoter; H4, H8, and H13 span the transcription region; and H15, H18, and H34 are at the intergenic spacer regions. **I**, **J** The rDNA occupancy of UBF is compromised in coronin 2B-deficient neurons. ChIP assay was performed on cortical neurons transduced with shCoro2B lentivirus or corresponding scramble control. Antibodies against UBF and mouse IgG as a control were used for rDNA immunoprecipitation. Precipitated DNA was assayed by qPCR using the primers shown in (**H**). The results are presented as the percentage of input values normalized to the control. Data were mean ± SEM (from three independent experiments; Mann–Whitney *U*-test in (**I**), unpaired Student’s *t*-test in (**J**); *p < 0.05, **p < 0.01, ***p < 0.001).

Given that coronin 2B aggregates in the nucleolus and colocalizes with UBF (a key component of the Pol I transcription pre-initiation complex), we subsequently investigated whether coronin 2B regulates rDNA transcription by facilitating the assembly of the transcription initiation complex at the rDNA promoter. Co-immunoprecipitation assay indicated that endogenous coronin 2B interacted with UBF in nuclear extracts derived from cortical neurons (Fig. 3A), which was confirmed by ectopic expression of coronin 2B in HEK293T cells (Fig. 3B, C). Given that the domain structure of coronin 2B has 7 repeated WD40 domains at its N-terminus followed by a unique region and a coiled-coil domain (UCC) at its C terminus (Supplementary Fig. 2A) [22], we overexpressed coronin 2B and its truncations (i.e., UCC and ΔUCC) in HEK293T cells. Co-immunoprecipitation assay further mapped the binding domain of coronin 2B with UBF and suggested that coronin 2B interacts with UBF via its 7 repeated WD40 domains (Supplementary Fig. 2B). Given that UBF forms a complex with SL-1 at the rDNA promoter region and recruits Pol I to form the transcriptional machinery [4, 6, 24], we examined whether coronin 2B participates in the organization of the rDNA transcriptional machinery via the UBF/SL-1 complex. Accordingly, the knockdown of coronin 2B in cortical neurons (Supplementary Fig. 3A, B) by lentivirus expressing short hairpin RNA (shRNA) against coronin 2B (shCoro2B) compromised the binding of both TAFIp110 (a subunit of SL-1 complex) and UBF to RPA194 (a subunit of Pol I) without altering their expressions (Fig. 3D–G). Taken together, these results suggest that coronin 2B is required to recruit Pol I to the rDNA promoter and thereby facilitates transcription initiation.

Next, we validated whether coronin 2B knockdown impairs the assembly of the transcription initiation complex by interfering with the binding of UBF to the rDNA promoter. Accordingly, we performed a chromatin immunoprecipitation (ChIP) assay followed by qPCR using primer pairs spanning the rDNA repeat (Fig. 3H). Consistent with a previous study [25], UBF was preferentially enriched in the promoter regions of rDNA (i.e., H42), moderately enriched in the transcribed regions (i.e., H4, H8, and H13), and rare in the untranscribed intergenic spacer regions (i.e., H15, H18, and H34) (Fig. 3I and Supplementary Fig. 4A). Of note, coronin 2B knockdown diminished the occupancy of UBF at the rDNA promoter and transcribed regions but not at the untranscribed intergenic spacer regions (Fig. 3J and Supplementary Fig. 4B). Thus, these results suggest that coronin 2B depletion reduces the promoter occupancy of UBF, which impairs the assembly of the transcription initiation complex.

### Coronin 2B depletion triggers nucleolar stress

**Fig. 4:**
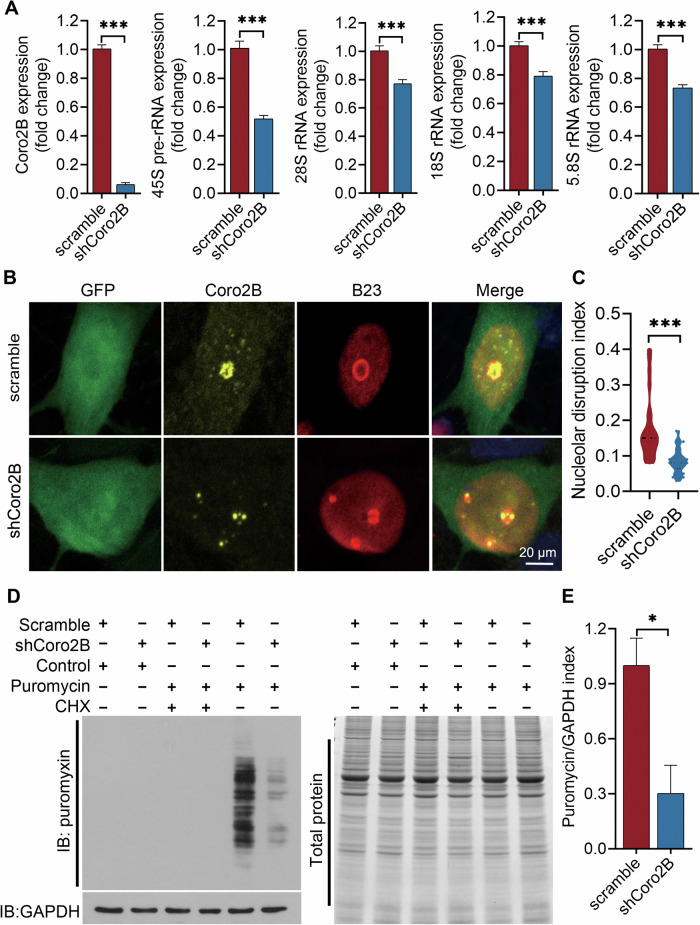
Coronin 2B knockdown triggers nucleolar stress. **A** Coronin 2B depletion decreases rDNA transcription in neurons. Cortical primary neurons cultured for 9 days were transduced with shCoro2B lentivirus or corresponding scramble control. RNA was extracted after 5 days, and the knockdown efficiency of coronin 2B and rRNA levels (i.e., 45S, 28S, 18S, and 5.8S) was examined by qPCR. Data are mean ± SEM (from three independent experiments; unpaired *t*-test with Welch’s correction, ***p < 0.001). **B***,* **C** Coronin 2B depletion impairs nucleolar integrity. Primary hippocampal neurons were cultured for 10 days and subjected to calcium phosphate transfection with coronin 2B shRNA or pSUPER vector. The cells were fixed and immunostained with anti-coronin 2B and anti-B23. The nuclei were stained with DAPI. Scale bar: 20 μm (**B**). Nucleolar integrity was quantified according to the nucleolar-to-nucleoplasmic ratio of average B23 fluorescence intensity. Data were mean ± SEM (from three independent experiments; Mann–Whitney *U-*test, ***p < 0.001) (**C**). **D**, **E** Coronin 2B depletion inhibits global protein synthesis. Cortical primary neurons transduced with shCoro2B lentiviral were treated with 5 μM puromycin and HEPES solvent control or cycloheximide (CHX) for 30 min. De novo protein synthesis was analyzed by western blot analysis and quantified according to the mean ratio of puromycin incorporation relative to total protein (i.e., Coomassie staining) (**E**). Data were mean ± SEM (from three independent experiments; unpaired Student’s *t*-test, *p < 0.05).

The perturbation of ribosome biogenesis or nucleolar structure triggers nucleolar stress. Specifically, the inhibition of rDNA transcription is a primary contributor to the induction of nucleolar stress [13]. Meanwhile, the nucleolar co-localization and interaction between coronin 2B and UBF raises the intriguing possibility that coronin 2B participates in rDNA transcription. Accordingly, we knocked down coronin 2B expression in cultured neurons with lentivirus expressing shCoro2B and examined rDNA transcription. Compared to neurons infected with the scramble control, the transcript levels of 45 S pre-rRNA as well as mature 28S, 18S, and 5.8S rRNA were significantly lower in coronin 2B-deficient neurons, suggesting that depletion of coronin 2B impairs rDNA transcription (Fig. 4A).

Loss of nucleolar B23 is a hallmark of nucleolar stress induced by the inhibition of rDNA transcription [13]. Therefore, we subsequently determined the distribution of B23 protein in the nucleoli of neurons by calcium phosphate transfection with shCoro2B or its scramble control. Coronin 2B knockdown dispersed the nucleolar distribution of B23. Meanwhile, in the control neurons, B23 remained in the nucleolus (Fig. 4B, C). These results suggest that coronin 2B-deficient neurons exhibit nucleoplasmic translocation of B23. In addition, we analyzed B23 staining in neurons treated with actinomycin D, which is commonly used to induce nucleolar stress via the inhibition of Pol I at low concentrations [26]. Similarly, upon actinomycin D administration, we observed neither B23 nor coronin 2B signal in neuronal nucleoli, suggesting nucleolar destruction (Supplementary Fig. 5). Thus, these results indicate that coronin 2B depletion inhibits rDNA transcription, destroys nucleolar integrity, and triggers nucleolar stress in neurons.

Given that rDNA transcription is the rate-limiting step of ribosome biogenesis that determines the global level of protein synthesis, we subsequently investigated whether coronin 2B depletion affects protein synthesis. Accordingly, we evaluated global protein synthesis by surface sensing of translation (SUnSET) [27] in cortical primary neurons infected with lentivirus expressing shCoro2B and detected puromycin incorporation into newly synthesized proteins using anti-puromycin antibody (12D10). Coronin 2B-deficient neurons exhibited significantly lower protein synthesis than the controls (Fig. 4D, E), suggesting that the inhibition of rDNA transcription caused by coronin 2B depletion impairs global protein synthesis. Hence, these results collectively indicate that coronin 2B knockdown impairs rDNA transcription, which triggers nucleolar stress and decreases global protein translation.

### Coronin 2B depletion shifts transcriptional activities toward neurodegeneration

**Fig. 5:**
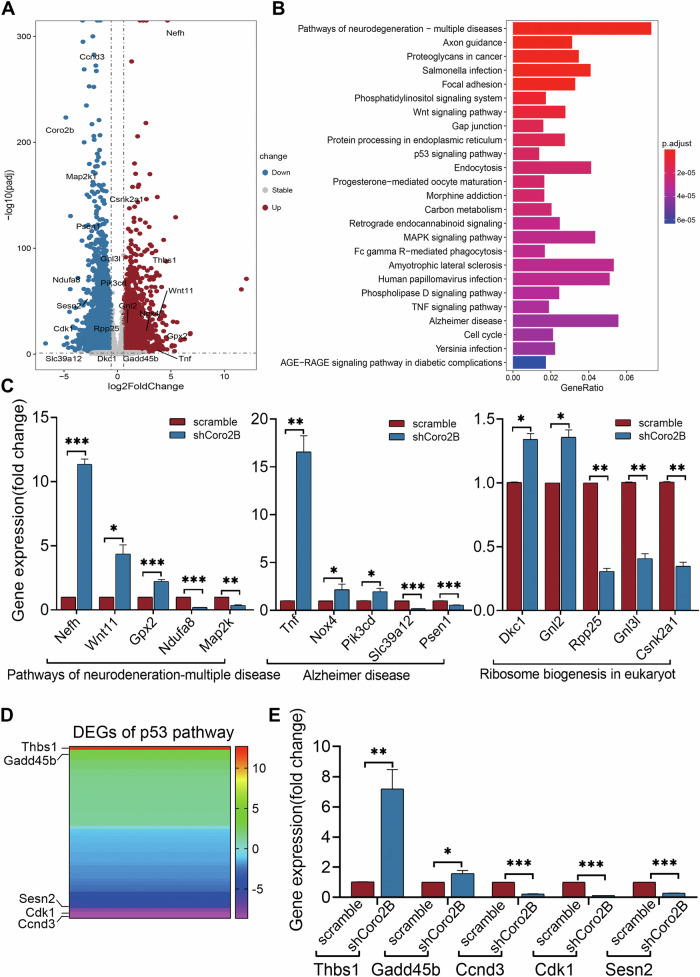
The neurodegeneration transcription program is activated upon coronin 2B depletion. **A** Volcano plot of differentially expressed genes (i.e., *p* < 0.01 and log_2_[fold-change] >1 or <−1). Vertical and horizontal dotted lines represent the threshold of −log_10_(adjusted *p* values) and log_2_(fold-change). **B** Kyoto Encyclopedia of Genes and Genomes (KEGG) pathway analysis of variable transcripts (cutoff: *p* < 0.01). **C** Validation of the expression levels of genes related to the indicated pathways from RNA sequencing analysis by qPCR. RNA was extracted from cortical primary neurons cultured for 9 days and transduced with shCoro2B lentivirus or corresponding scramble control. Then, qPCR was performed using primers for selected genes. Data were mean ± SEM (from three independent experiments; unpaired *t*-test with Welch’s correction; *p < 0.05, **p < 0.01, ***p < 0.001). **D** Heatmap of p53 pathway-related gene expression from RNA sequencing analysis**. E** RT-qPCR analysis of the top five genes with the greatest variable expression in the p53 pathway. Data were mean ± SEM (from three independent experiments; unpaired *t*-test with Welch’s correction; *p < 0.05, **p < 0.01, ***p < 0.001).

To investigate how coronin 2B-mediated nucleolar stress affects gene expression, we performed RNA sequencing (RNA-seq) analysis on cortical neurons infected with lentivirus expressing shCoro2B or scramble control. Principal component analysis divided the RNA-seq data into two distinct clusters that reflect the alteration of gene expression in coronin 2B-deficient neurons compared with the controls (Supplementary Fig. 6A). Furthermore, transcriptome analysis revealed 5440 differentially expressed genes (cutoff: *p* < 0.01 and log_2_[fold-change] >1 or <−1) in coronin 2B-deficient neurons, including 2563 upregulated and 2877 downregulated genes (Fig. 5A and Supplementary Fig. 6B; see also Supplementary Table 1). Kyoto Encyclopedia of Gene and Genomes (KEGG) pathway analysis identified the 25 most enriched signaling pathways (cutoff: *p* < 0.01) (Fig. 5B). Most of these 25 pathways are associated with neurodegeneration or apoptosis, including “neurodegeneration-multiple diseases,” “axon guidance,” “Wnt signaling pathway,” “p53 signaling pathway,” and “Alzheimer’s disease,” suggesting that coronin 2B is involved in the regulation of neuronal degeneration and apoptosis. Furthermore, the ribosome biogenesis pathway was significantly altered in coronin 2B-deficient neurons (*p* < 0.001, Supplementary Table 1), which is consistent with the above results and further suggests that coronin 2B knockdown results in rDNA transcription inhibition and nucleolar destruction. In addition, the main terms in Gene Ontology (GO) enrichment analysis of the differentially expressed genes (cutoff: *p* < 0.01) were involved in neuronal survival and synaptic transduction, including “DNA repair,” “autophagy,” “process utilizing autophagic mechanism,” “neuron to neuron synapse,” “postsynaptic specialization,” “synaptic membrane,” and “GTPase regulator activity” (Supplementary Fig. 6C). To confirm the reliability of RNA-seq analysis, we performed qPCR to validate the differentially expressed genes in enriched KEGG pathways related to neurodegeneration and apoptosis. Accordingly, the results of RNA-seq and qPCR were highly consistent (Fig. 5C). Taken together, these results indicate that coronin 2B-mediated nucleolar stress activates the transcription program toward neurodegeneration and apoptosis.

### Coronin 2B deficiency-induced nucleolar stress stabilizes p53

**Fig. 6:**
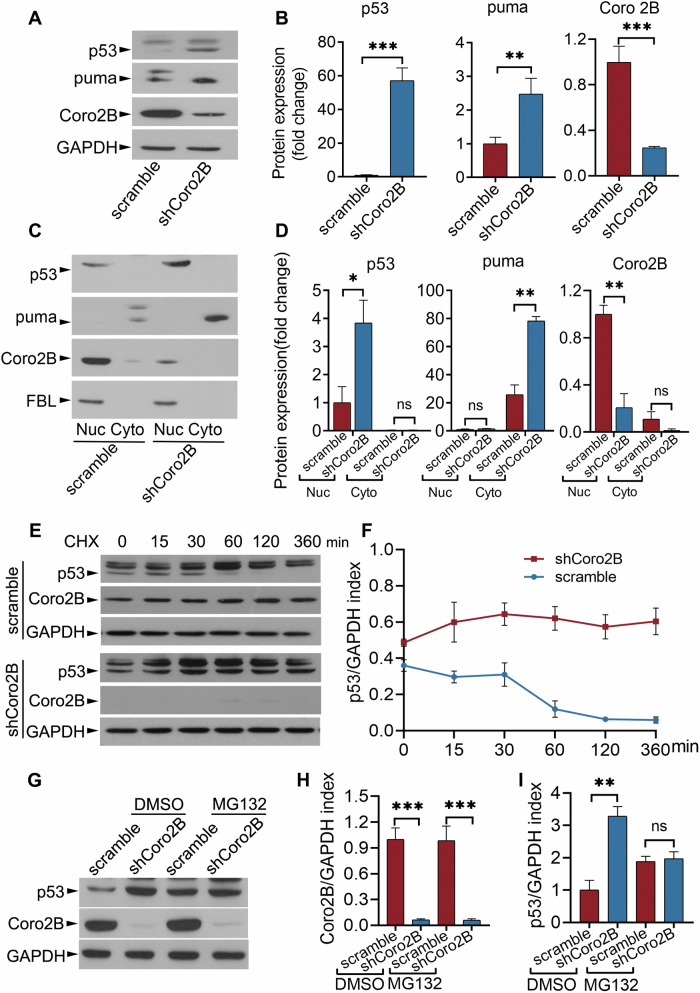
Coronin 2B depletion accelerates p53 accumulation and activation. Cortical neurons cultured for 9 days were transduced with shCoro2B lentivirus or corresponding scramble control. After 5 days, total protein (**A**), as well as cytoplasmic and nuclear fractions (**C**), were collected, and protein expression was detected by western blot analysis with the indicated antibodies and normalized to GAPDH (for total protein) or FBL (for nucleolar protein). The quantification is shown in (**B**) and (**D**), respectively. Data were mean ± SEM (from three independent experiments; unpaired *t*-test with Welch’s correction in (**B**), unpaired Student’s *t*-test in (**D**); *p < 0.05, **p < 0.01, ***p < 0.001). **E**–**I** Cortical neurons transduced with shCoro2B lentivirus were treated with 50 µg/mL cycloheximide (CHX) for the indicated periods (**E**) or 20 µM MG132 for 6 h (**G**). The cell lysates were subjected to western blot analysis using the indicated antibodies and normalized to that of GAPDH. The quantification is shown in (**F**, **H**, **I**). Data were mean ± SEM (from three independent experiments; unpaired *t*-test with Welch’s correction; *p < 0.05, **p < 0.01, ***p < 0.001, ns not significant).

The tumor suppressor, p53, is one of the most critical factors for eliciting the downstream effects of nucleolar stress, such as cell cycle arrest and apoptosis [15]. Under physiological conditions, p53 is a short-lived protein that is almost undetectable because it is quickly ubiquitinated by the E3 ubiquitin ligase, MDM2, followed by its subsequent degradation via a proteasome-dependent pathway. In response to diverse stressors, p53 rapidly accumulates in nuclei and becomes activated [15]. Given the enrichment of the p53 pathway in our RNA-seq data and the key roles of p53 in nucleolar stress, we subsequently examined the effects of coronin 2B depletion on the p53 pathway. Consistent with the RNA-seq data (Fig. 5D), coronin 2B depletion altered the transcription levels of p53 target genes in neurons, including *Thubs1*, *Gadd45*, *Ccnd3*, *Cdk1*, and *Sesn2* (Fig. 5E), suggesting activation of p53 transcription activity. Indeed, the protein levels of p53 and its downstream target protein, puma, were elevated in coronin 2B-deficient neurons (Fig. 6A–D), suggesting that coronin 2B knockdown leads to the accumulation of p53 protein and the induction of its transcription activities.

To clarify whether the elevated p53 protein level in coronin 2B-deficient neurons is due to protein stabilization, transcriptional regulation, or translational regulation, we measured *p53* mRNA levels by qPCR. The steady-state mRNA level of p53 remained constant in neurons regardless of coronin 2B knockdown (Supplementary Fig. 7). We then performed a chase experiment on cortical neurons infected with lentivirus expressing shCoro2B for 5 days by treating them with the protein synthesis inhibitor, cycloheximide (CHX). Quantification of p53 protein levels showed that coronin 2B knockdown delayed the degradation of p53 (Fig. 6E, F). Furthermore, administration of the proteasomal inhibitor, MG132, showed that the delayed degradation of the p53 protein was due to the inhibition of proteasome-mediated degradation (Fig. 6G–I). Therefore, these results indicate that the increased p53 protein level upon coronin 2B knockdown is due to p53 protein stabilization and not increased synthesis.

**Fig. 7:**
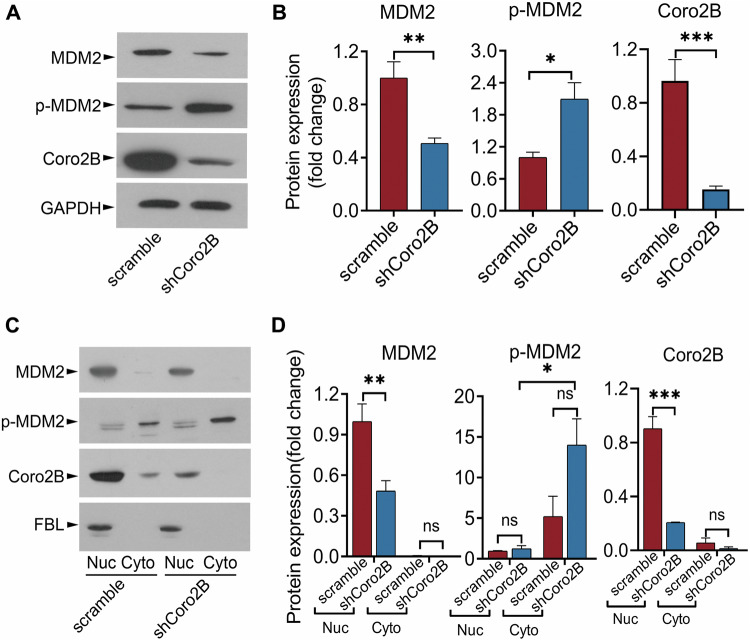
Coronin 2B depletion accelerates MDM2 phosphorylation and degradation. Cortical neurons at 9 days in vitro (DIV) were treated with shCoro2B lentivirus or scramble control. Total protein (**A**), as well as cytoplasmic and nuclear fractions (**C**), were collected, and protein expression was detected by western blot with the indicated antibodies and normalized to that of GAPDH (for total protein) or FBL (for nucleolar protein). The quantification is shown in (**B**, **D**). Data were mean ± SEM (from three independent experiments; unpaired Student’s *t*-test in (**B**), Mann–Whitney *U-*test in (**D**); *p < 0.05, **p < 0.01, ***p < 0.001, ns not significant).

Coronin 2B-deficient neurons also exhibited a reduced total protein level of MDM2 and increased phosphorylation of MDM2 at serine 166 (Fig. 7A, B). As phosphorylated MDM2 is restricted to the cytoplasm, it is unable to bind to p53 in the nucleus [28]. Accordingly, the nucleocytoplasmic fraction experiment showed that phosphorylated MDM2 was greatly enriched in the cytosolic fraction in coronin 2B-deficient neurons accompanied by a reduced level in the nucleus (Fig. 7C, D). This further suggests that coronin 2B knockdown prevents MDM2 from entering the nucleus, which results in the nuclear accumulation of p53. Thus, these results collectively indicate that the decrease of MDM2 in the nucleolus is advantageous for the accumulation of p53 in coronin 2B-deficient neurons.

### Coronin 2B-mediated nucleolar stress induces neuronal apoptosis

**Fig. 8:**
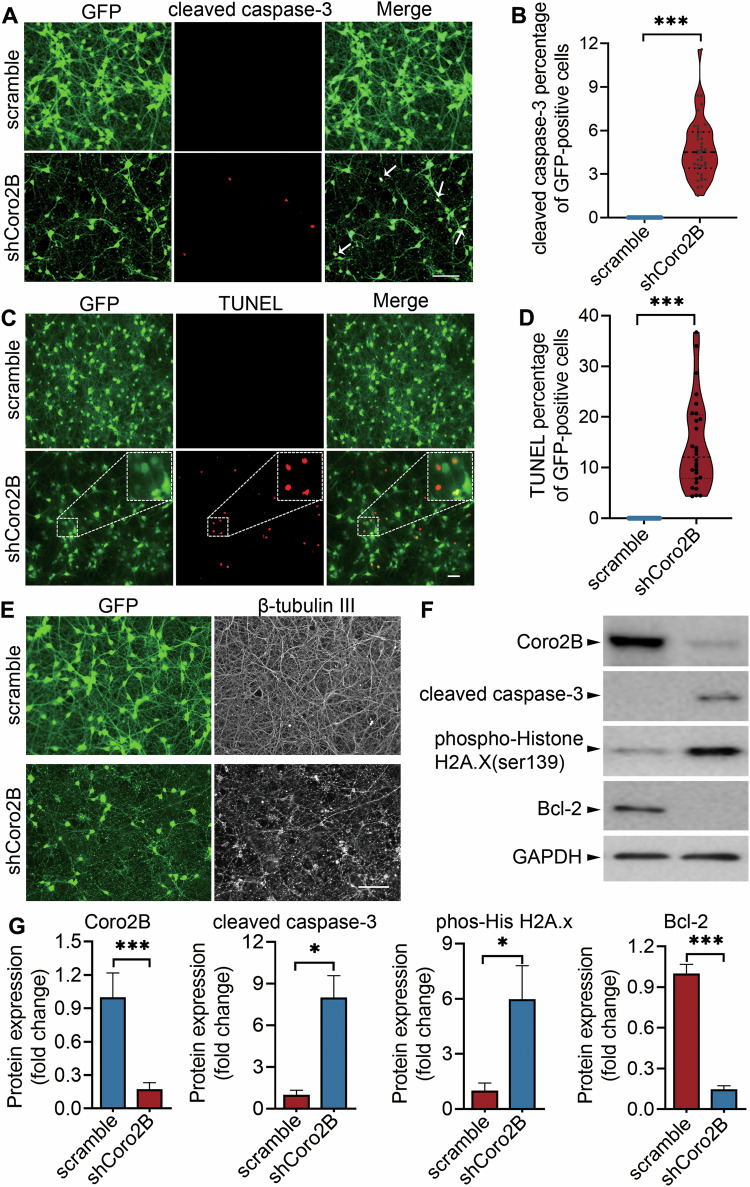
Coronin 2B knockdown leads to neuronal apoptosis. Cortical neurons were cultured for 9 days and infected with lentivirus expressing shCoro2B or corresponding scramble control. After 5 days, the cells were fixed and immunofluorescence staining was performed for cleaved caspase-3 (**A**), TUNEL assay (**C**), and β-tubulin III assay (**E**). Cleaved caspase-3 (**B**) and TUNEL (**D**) signals were quantified by immunostaining and normalized to the number of GFP-positive cells. At least 100 GFP-positive cells were counted for each condition. Data were mean ± SEM (from three independent experiments; Mann–Whitney *U-*test, ***p < 0.001). Scale bar: 100 μm. **F** Cortical neurons cultured for 9 days were transduced with shCoro2B lentivirus or corresponding scramble control. After 5 days, total protein was collected, and protein expression was detected by western blot analysis with the indicated antibodies. The quantification is shown in (**G**). Data were mean ± SEM (from three independent experiments; unpaired Student’s *t*-test; *p < 0.05, **p < 0.01, ***p < 0.001).

One of the ultimate consequences of nucleolar stress is the initiation of apoptosis [13]. Therefore, we subsequently investigated the effect of nucleolar stress induced by coronin 2B depletion on neuronal apoptosis. Caspases are cellular mediators of apoptosis that exist as inactive precursors activated by proteolytic cleavage. In particular, cleaved caspase-3 is a hallmark of the initiation of apoptosis [29]. Therefore, we measured the levels of cleaved caspase-3 in coronin 2B-deficient neurons. There was significantly more cleaved caspase-3–positive neurons among coronin 2B-deficient neurons, whereas the immunostaining signal of cleaved caspase-3 was barely detectable in the controls (Fig. 8A, B). TUNEL (terminal deoxynucleotidyl transferase dUTP nick-end labeling) assay detects apoptotic cells that have undergone extensive DNA degradation during the late stages of apoptosis [30]. Here, we found that the knockdown of coronin 2B led to a significant increase in TUNEL labeling compared with the scramble control (Fig. 8C, D). In addition, shCoro2B-expressing neurons formed numerous β-tubulin III puncta, indicating that the neurons underwent degeneration, as their microtubes had disassembled (Fig. 8E). Furthermore, we examined the expression levels of several typical apoptosis-associated proteins, including cleaved caspase-3, Bcl-2, and phosphorylated histone H2A.X, in coronin 2B-deficient neurons by western blot analysis. Consistent with our findings above, coronin 2B-knockdown neurons exhibited elevated levels of pro-apoptotic markers, such as cleaved caspase-3 and phosphorylated histone H2A.X, alongside a decreased level of the anti-apoptotic marker, Bcl-2 (Fig. 8F, G). Taken together, these results indicate that coronin 2B-mediated nucleolar stress induces neuronal apoptosis.

## Discussion

**Fig. 9:**
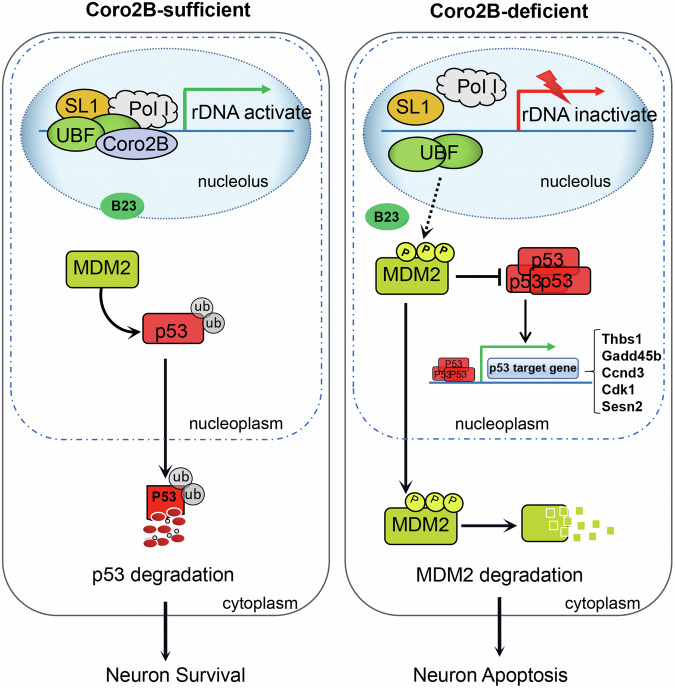
Schematic diagram of the roles of coronin 2B in the neuronal nucleolus. Under physiological conditions, coronin 2B localizes at the nucleolus in neurons, where it interacts with UBF and promotes rDNA transcription. MDM2 freely ubiquitinates p53, thereby promoting its degradation. Meanwhile, coronin 2B knockdown impairs the formation of the transcription initiation complex, thereby repressing rDNA transcription. MDM2 is phosphorylated and escapes into the cytoplasm. Furthermore, p53 accumulates and is activated, which subsequently leads to neuronal apoptosis.

The progressive degeneration or apoptosis of specific neurons in the central or peripheral nervous system contributes to neurodegenerative diseases, including Alzheimer’s disease, Parkinson’s disease, and amyotrophic lateral sclerosis (ALS). Although the vulnerability of neurons varies by disease, impaired nucleolar activity is a common phenomenon during neurodegeneration [1, 13]. Therefore, identifying novel rDNA transcriptional regulators and understanding their specific regulatory mechanisms will contribute to the development of nucleolar transcription activity-targeting therapeutic strategies for neurodegeneration. In the present study, we identified a novel role of the nucleolar protein, coronin 2B, wherein it specifically localizes at the nucleolus in neurons and interacts with UBF, a key component of the rDNA transcription pre-initiation complex. Meanwhile, we found that coronin 2B deficiency inhibits rDNA transcription, destroys nucleolar integrity, and triggers nucleolar stress, which ultimately leads to p53 activation and neuronal apoptosis (Fig. 9). Thus, our results suggest that coronin 2B is a novel nucleolar regulator involved in the maintenance of neuronal survival.

### Regulation of rDNA transcription by coronin 2B in neurons

As a member of the high mobility group box protein family [31], UBF orchestrates the assembly of the transcription pre-initiation complex and establishment of active rDNA chromatin, which performs distinct roles in transcription initiation, chromatin remodeling, gene regulation, and epigenetic programming [32, 33]. Here, we showed that coronin 2B specifically localizes at the UBF-containing regions of the nucleolus in neurons and coordinates with UBF for the docking and assembly of the rDNA transcription pre-initiation complex. The results of nucleocytoplasmic fractioning (Fig. 1A–D) and the immunostaining of coronin 2B in neurons by STORM (Figs. 1E, F, 2) consistently show the localization of coronin 2B in neuronal nuclei. As a member of the type II coronin family, coronin 2B exhibits significant homology with another family member, coronin 2A, which similarly localizes within nuclei. Moreover, coronin 2A can serve as a component of the NcoR complex in nuclei to regulate transcription in immune cells [34]. We and others have shown that coronin 2B can form oligomers via its coiled-coil domain to provide a large platform for protein–protein interactions through its seven repeated WD40 domains [21–23]. Thus, the key role of coronin 2B in the nucleolus may be to act as a molecular scaffold to facilitate the assembly of Pol I transcriptional machinery via interaction with UBF. Recent studies support the notion that the nucleolus is a multiphase biomolecular condensate whose formation by liquid–liquid phase separation facilitates the initial steps of ribosome biogenesis [35, 36]. Accordingly, proteins with a coiled-coil domain are sufficient to drive liquid–liquid phase separation via both polymeric and multimeric multivalency mechanisms [37]. The phase separation of the fibrillary center and dense fibrillary component, where Pol I transcription occurs [38], contributes to the efficient transcription of Pol I [36]. Therefore, as a structural protein, coronin 2B may also act as a nucleolar regulator that controls the biophysical properties of the fibrillary center/dense fibrillary component to regulate rDNA transcription. In addition, UBF activity is reported to be regulated by post-translational modifications. For example, in vigorously dividing cells, UBF is phosphorylated at multiple sites, which enhances its associations with SL-1 and Pol I [39]. Besides phosphorylation, the acetylation of UBF promotes the association of Pol I with the rDNA promoter [40]. Nevertheless, further research is required to determine whether the compromised occupancy of UBF at the rDNA promoter upon coronin 2B depletion depends on such post-translational modifications.

### Nucleolar stress and p53 activation in coronin 2B-deficient neurons

The p53 protein plays a major role in maintaining cellular homeostasis by responding to various internal and external stressors. Under physiological conditions, the E3 ubiquitin ligase, MDM2, mediates p53 ubiquitination and degradation, keeping the p53 level low. However, in response to nucleolar stress, several nucleolar proteins, including RPL5 [41], RPL11 [42], and RPL23 [43], are released into the nucleoplasm where they bind to MDM2, inhibiting its E3 ubiquitin ligase activity. Thus, further studies are required to define the key upstream triggers of p53 activation elicited by coronin 2B-mediated nucleolar stress. MDM2 protein is reported to be quickly auto-ubiquitinated and degraded, thereby allowing p53 to accumulate and become activated in response to DNA damage [44]. Here, we observed that coronin 2B knockdown leads to MDM2 phosphorylation and decreased MDM2 protein level. Therefore, the pool of MDM2 available to ubiquitinate p53 is insufficient to degrade p53, resulting in its accumulation in the nucleolus.

Phosphorylation is an important post-translational modification that regulates p53–MDM2 interaction. Phosphorylation of MDM2 in close proximity to its RING domain, which is responsible for ubiquitinating and degrading p53, consequently inhibits its ability to ubiquitinate p53; instead, this promotes the self-ubiquitination and proteasomal degradation of MDM2 [28, 45]. In contrast, phosphorylation of MDM2 at serine 166 and 186 in its RxRxxS/T motifs, a region proximal to the nuclear localization signal (i.e., amino acids 181–185) and nuclear export signal (i.e., amino acids 190–200), promotes the translocation of MDM2 from the cytoplasm to the nucleus, thereby increasing p53 degradation [46, 47]. In the present study, coronin 2B knockdown led to MDM2 phosphorylation at serine 166, thereby promoting MDM2 translocation into the cytoplasm but not the nucleolus. However, it is unknown whether the different outcomes of MDM2 phosphorylation at different sites are due to differences in cellular contexts or physiological conditions. In addition, given that nucleolar stress-induced p53 stabilization and activation are involved in the interactions of many nucleolar proteins with p53/MDM2 and are regulated by numerous post-translational modifications, an unknown mechanism may underlie the translocation of MDM2 in neurons.

### Inhibition of rDNA transcription triggers neuronal apoptosis

Ribosome biogenesis is one of the most energy-intensive processes in cells, consuming approximately 80% of cellular energy [48, 49]. As a result, the neuroprotective reduction of nucleolar transcription could contribute to dementia by promoting the loss and/or dysfunction of synapses. Therefore, it is of interest to investigate whether enhancing nucleolar transcription activity can alleviate neuronal death in neurodegenerative diseases. Of note, nucleolar stress also occurs in neurodegenerative diseases [13, 14, 50]. For example, hippocampal brain tissue from patients with Alzheimer’s disease exhibits greater dysregulation of nucleolar activity and alteration of protein synthesis than tissues from patients with mild cognitive impairment [51, 52]. Patients with Alzheimer’s disease also exhibit decreased expression of 18S and 28S rRNA in the parietal cortex [53] and peripheral blood [54, 55]. Moreover, in Parkinson’s disease, nucleolar volume and rDNA transcription are decreased in dopaminergic neurons, and the disruption of nucleolar function is inversely correlated with disease duration [56, 57]. These lines of evidence collectively imply that nucleolar activity is tightly regulated to sustain neuronal functions, including neuronal survival and synaptic transmission.

Although the role of coronin 2B in human disease has not been extensively documented, other coronin family members are implicated in various human diseases, including immune system disorders [58, 59], diffuse gliomas [60], neuronal behavior disorders [61, 62], cancers [63], and obesity [64, 65]. Here, we identified the critical role of coronin 2B during nucleolar stress and its regulation of neuronal apoptosis. Our findings suggest potential implications of neuronal nucleolar stress in various human diseases, particularly in degenerative conditions involving elevated cell apoptosis. Indeed, nucleolar stress occurs in several neurodegenerative diseases, including ALS [66], frontotemporal dementia [66], and Parkinson’s disease [56, 57]. Therefore, maintaining proper nucleolar function and alleviating the stress signals emanating from neuronal nucleoli may represent a novel therapeutic approach for protecting neurons from cell death and preserving functional neural circuits. Nevertheless, further experiments are required to clarify the specific molecular mechanisms and biological consequences of neurodegeneration-associated nucleolar stress.

## Materials and methods

### Reagents and constructs

Antibodies against coronin 2B (1:1000 dilution; HPA017960), GAPDH (1:5000 dilution; G8795), and β-tubulin III (1:500 dilution; T8578) were purchased from Sigma-Aldrich (St. Louis, MO, USA). Antibodies against HA (1:2000 dilution; sc-805), MDM2 (1:1000 dilution; sc-965), p53 (1:1000 dilution; sc-99), RPA194 (1:1000 dilution; sc-48385), and UBF (1:500 dilution; sc-13125) were purchased from Santa Cruz Biotechnology (St. Louis, MO, USA). Anti-puromycin antibody (1:500 dilution; 12D10) was purchased from Merck Millipore (Billerica, MA, USA). Antibodies against B23 (1:1000 dilution; 32-5200) and FBL (1:1000 dilution; 480009) were purchased from Life Technologies (Carlsbad, CA, USA). Antibodies against puma (1:1000 dilution; 24633), phospho-MDM2 (1:1000 dilution; Ser166, 3521), and cleaved caspase-3 (1:500 dilution; Asp175, 9661 L) were purchased from Cell Signaling Technology (Danvers, MA, USA). Secondary antibodies included goat anti-rabbit and goat anti-mouse IgG (both 1:2000 dilution).

The full-length coronin 2B (Coro2B) gene with an HA tag was cloned into the pcDNA3.0 vector. Then, shRNAs against coronin 2B (5′-GAGGATCTGTCCATGCCAA-3′) or its corresponding scramble sequence (5′-GCAGTTAGTGAACGCAGTC-3′) synthesized by Life Technologies [22] were cloned into the pSUPER and pFUGW vectors for calcium phosphate transfection and lentivirus transduction, respectively. HEK293T cells were used to package lentivirus particles with packaging plasmids (pDelta8.74, pMD2.G). Lentiviral backbone and packaging plasmids were transfected into HEK293T cells using polyethyleneimine. Lentivirus-containing media were harvested after 48 h and filtered through a 0.45-μm filter to remove cellular debris. The virus was concentrated using an Optima XPN-100 ultracentrifuge (Beckman) and resuspended in D-PBS.

### Culture and transfection of primary rat neurons

Hippocampal neurons were dissociated from Sprague–Dawley rat embryos at embryonic day 18 and cultured at 1 × 10^5^ cells per 18-mm coverslip coated with poly-d-lysine (1 mg/mL). After 10 days, the hippocampal neurons were subjected to calcium phosphate transfection with coronin 2B shRNA or scramble control vector. The cortical neurons were seeded on a 60-mm dish coated with poly-l-lysine (0.1 mg/mL) for 9 days and subsequently transduced with lentivirus expressing shCoro2B or corresponding scramble shRNA as a negative control. The neurons were cultured in Neurobasal Medium (Life Technologies) containing 2% (v/v) B27 supplement (Life Technologies) at 37 °C in a 5% CO_2_ humidified atmosphere for 14 days for subsequent experiments.

### Immunofluorescence assay

Hippocampal neurons were grown on coverslips for 14 days before being fixed with 4% paraformaldehyde in D-PBS for 15 min at room temperature. The cells were washed three times with D-PBS, permeabilized with 0.4% Triton X-100, and blocked with 1% PBS-normal goat serum for 30 min. Immunostaining was performed by incubation with primary antibodies overnight at 4 °C followed by incubation with Alexa Fluor-conjugated secondary antibodies for 1–2 h at room temperature. The neurons were stained with DAPI and mounted with ProLong Glass antifade mountant (Thermo Fisher Scientific). Images were captured by confocal microscopy (Zeiss LSM 880). For the nucleolar integrity assay, the average fluorescence intensity of B23 protein in the nucleoli and nucleoplasm was measured using ImageJ software (version 1.53 S). The ratio of the fluorescence intensity of the nucleoli to the nucleoplasm was used as an indicator of nucleolar disruption. For the TUNEL assay, the cells were fixed and apoptotic signals were detected using a One Step TUNEL Apoptosis Assay Kit (Beyotime, Jiangsu, China). Total number of apoptotic cells were determined by counting the number of TUNEL-labeled cells among the GFP-positive cells.

### Co-immunoprecipitation and western blot analysis

Nuclear extracts from cortical neurons were obtained using Nuclear and Cytoplasmic Extraction Reagent (Thermo Fisher Scientific) supplemented with protease inhibitor cocktail. Protein concentration was determined using a BCK kit (Pierce Biotechnology). Cell lysate (1 mg protein) was diluted twice to reduce the concentration of salt hydronium, incubated with primary antibodies overnight at 4 °C, and then incubated with 30 µL protein G beads for 1–2 h at 4 °C. After washing three times with lysis buffer, the samples were resuspended in SDS loading buffer. Co-immunoprecipitated proteins were detected by western blot analysis using specific antibodies. HEK293T cells were transfected with pcDNA3.0-Coro2B-HA for 24 h and harvested in lysis buffer (50 mM Tris-Cl [pH 8.0], 100 mM NaCl, 50 mM NaF, 10% glycerol, 2 mM EGTA, and 0.4% NP-40) and protease inhibitors. After swirling gently for 30 min at 4 °C, the protein lysates were centrifuged at 12,000 rpm for 15 min at 4 °C. The supernatants were collected, incubated with primary antibodies, and submitted to western blot analysis.

### Chromatin immunoprecipitation assay

Cortical neurons were fixed in 1% formaldehyde (Sigma-Aldrich, F1635) for 11 min at room temperature and quenched with glycine to a final concentration of 0.125 M for 5 min with slow shaking. The cells were harvested and lysed in cell lysis buffer (10 mM Tris-Cl [pH 8.0], 10 mM NaCl, and 0.5% NP-40). Chromatin was sonicated using a Digital Sonifier (Branson) switched ON for 30 s and OFF for 30 s for 25 cycles to obtain ~200–500-bp chromatin fragments. Sheared chromatin was incubated with prepared antibody–bead complex and rotated overnight at 4 °C. The beads were washed twice with RIPA-150 (50 mM Tris-HCl [pH 8.0], 0.15 M NaCl, 1 mM EDTA [pH 8.0], 1% SDS, 1% Triton X-100, and 0.1% sodium deoxycholate), twice with RIPA-500 (50 mM Tris-HCl [pH 8.0], 0.5 M NaCl, 1 mM EDTA [pH 8.0], 1% SDS, 1% Triton X-100, and 0.1% sodium deoxycholate), once with LiCl wash buffer (50 mM Tris-Cl [pH 8.0], 1% NP-40, 0.7% sodium deoxycholate, 0.25 M LiCl_2_, and 1 mM EDTA [pH 8.0]), and once with TE buffer (10 mM Tris-Cl [pH 8.0] and 1 mM EDTA [pH 8.0]). The immuno-bound chromatin was eluted from the beads in elution buffer (1% SDS and 100 mM NaHCO_3_) for 4 h at 65 °C with shaking at 2000 rpm every 15 min. The eluted material was treated with RNase A for 2 h at 37 °C followed by Proteinase K for 2 h at 55 °C. DNA was extracted with phenol:chloroform:isoamyl alcohol purified using a QIAquick PCR purification kit (Qiagen). Immunoprecipitated DNA and input DNA were analyzed by RT-qPCR using primer pairs specific to the rDNA repeat (Supplementary Table 2). The ChIP-qPCR results are presented as the percentage of input DNA (% input).

### RNA sequencing analysis

Primary cortical neurons were cultured for 9 days and transduced with lentivirus expressing shCoro2B or the corresponding scramble control. After 5 days, total RNA was extracted using TRIzol reagent (Thermo Fisher Scientific). The cDNA library was produced on an Illumina HiSeq Platform and subjected to quality control with paired-end reads. Quality control on raw reads was performed with FastQC (version 0.11.9). Adapter trimming, the cutting of the first 12 bases, and the removal of trimmed reads shorter than 50 bp were performed using Trimmomatic (version 0.39). Trimmed reads were mapped to the Ensembl *Rattus norvegicus* rn6 genome (version 0.13.0). STAR (version 2.7.9a) was used to align reads. RSEM (version 1.2.28) was used to obtain counts and fragments per kilobase per million mapped fragments (FPKM). Differentially expressed genes were analyzed using the *DESeq2* package in R. Genes with a false discovery rate set at an adjusted *p* value <0.01 and log_2_(fold-change) >1 or <−1 were considered differentially expressed genes. GO and KEGG pathway analysis were performed using the *clusterProfiler* package in R, and heatmaps were generated using the *ComplexHeatmap* package in R. The representative differentially expressed genes were validated by quantitative PCR (qPCR). Primers for qPCR are listed in Supplementary Table 2. The RNA-seq datasets generated by this study have been deposited to the NCBI Sequence Read Archive under BioProject number PRJNA1111676.

### Super-resolution imaging

A Nanoimager S microscope (Oxford Nanoimaging) was used for dSTORM imaging. Briefly, fixed cells were treated with 0.1% NaBH_4_ to reduce background fluorescence, immunostained, and then immersed in dSTORM buffer (BCubed Reagent Kit, Oxford Nanoimaging) for imaging. During the 2-color dSTORM experiments, the light was collected using a 100× oil-immersion objective (numerical aperture: 1.4). The signal for coronin 2B was acquired first using a 640-nm laser; the signals for B23, UBF, and FBL were then acquired using a 561-nm laser. To minimize photobleaching, the focal plane of the protein was found under the 640-nm laser at 8% power; the laser was then switched to the 561-nm laser at 5% power for image acquisition. For all images, 10,000 frames were recorded with an exposure time of 10 ms. Next, dSTORM images were analyzed and rendered using NimOS (version 1.19, Oxford Nanoimaging) and CODI, a cloud-based platform for single-molecule fluorescence microscopy data analysis (https://alto.codi.bio/).

### Statistical analysis

All experiments were performed independently at least three times. Data were expressed as mean ± SEM. Statistical analysis was performed using GraphPad Prism 8. The distribution of data was tested for normality. If the assumption for normality could not be rejected, differences between two groups were analyzed using unpaired Student’s *t*-tests or unpaired *t*-test with Welch’s correction, depending on the *F*-test results for variance comparison. If the assumption for normality was rejected, the significance of differences was analyzed using the Mann–Whitney *U*-test. The criterion for significance was set at *p* < 0.05.

### Supplementary information

Supplementary Figures

Supplementary Table 1

Supplementary Table 2

Supplementary Table 3

Uncropped Western blots

## Data Availability

All study data were available upon reasonable request from the corresponding authors.
