# Supplementary material for: Coronin 2B deficiency induces nucleolar stress and neuronal apoptosis
Source: Cell Death Dis. 2024 Jun 27;15(6):457. doi: 10.1038/s41419-024-06852-x (PMC11211331; doi:10.1038/s41419-024-06852-x)
Supplement: Supplementary file 1 — Supplementary Figures [file 41419_2024_6852_MOESM1_ESM.pdf]

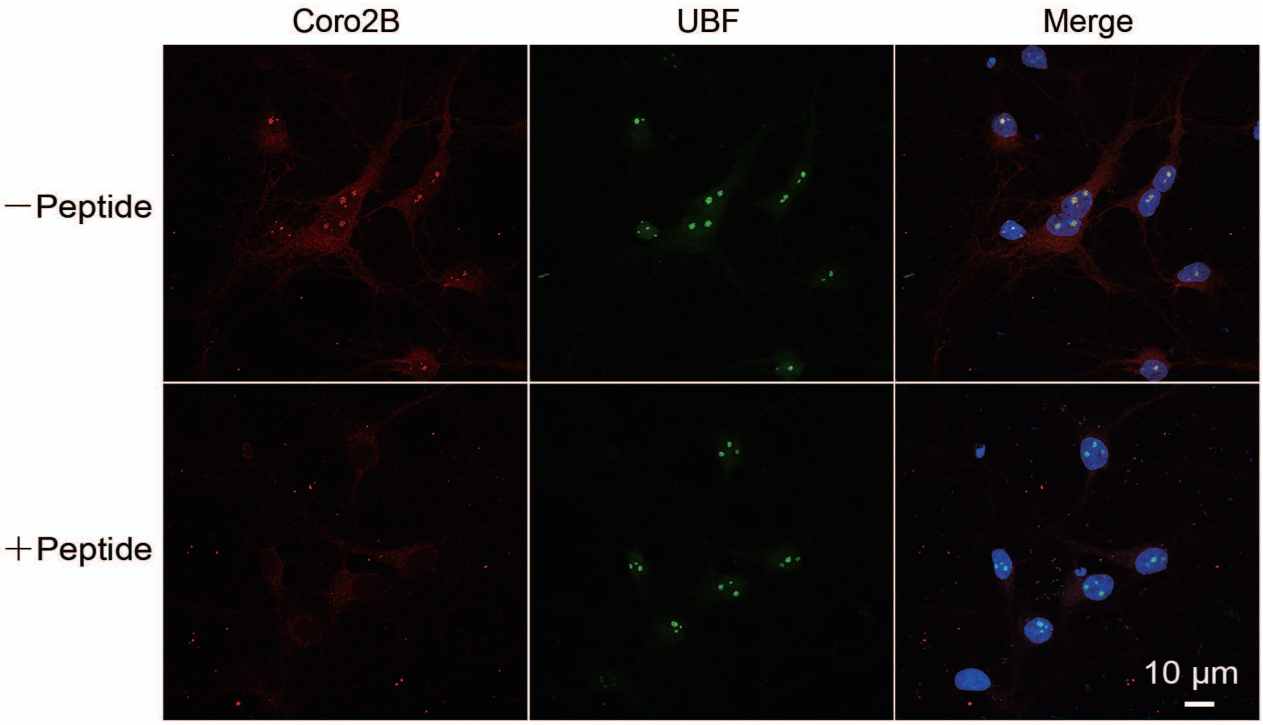

Supplementary Figure 1. Antigen peptide completely neutralizes anti-coronin 2B antibody. Fixed cultured hippocampus neurons were co-immunostained with anti-UBF (green) and anti-coronin 2B (red) antibodies with or without neutralization with antigen peptide. Scale bar: 10 μm.

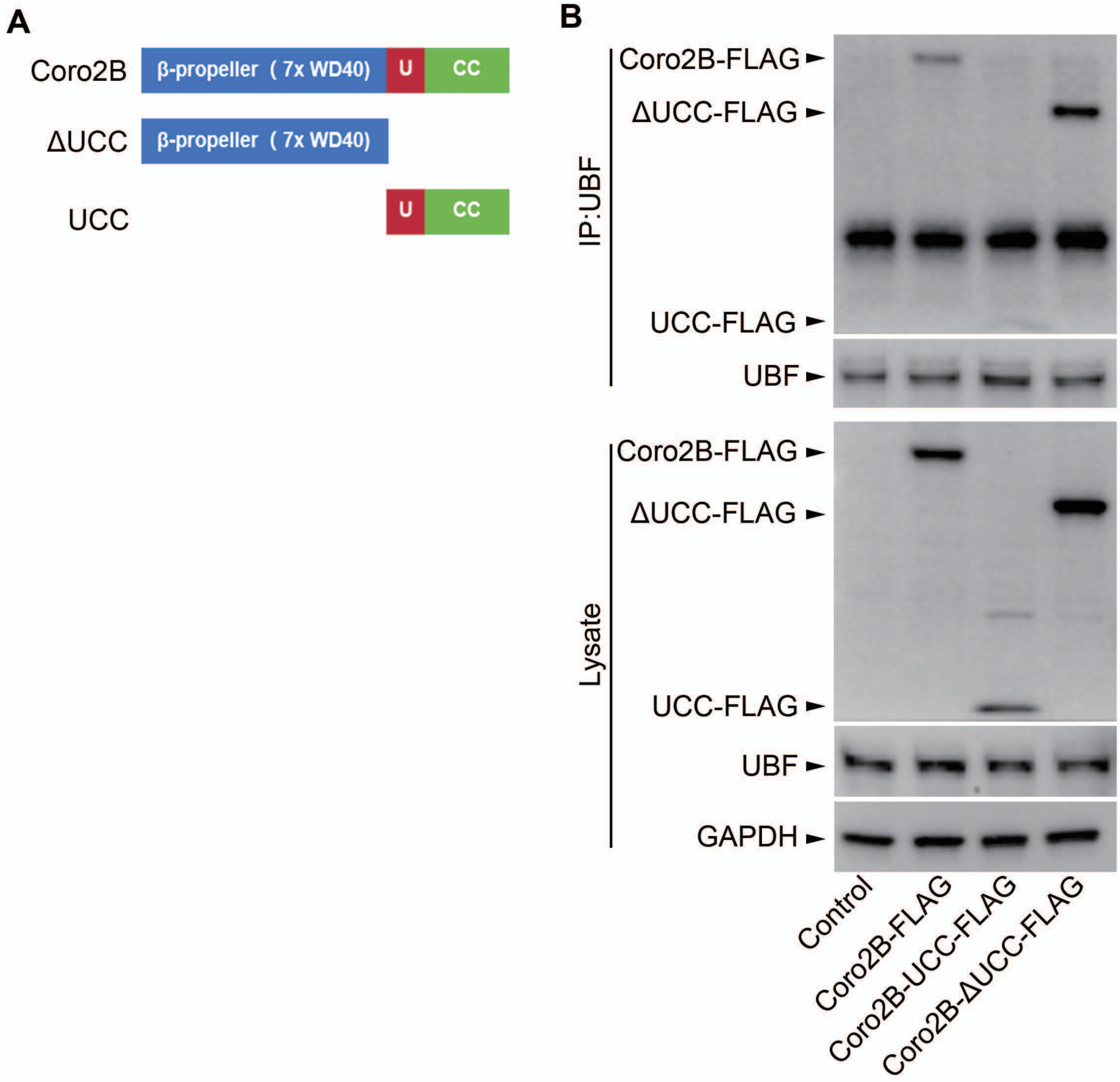

Supplementary Figure 2. Mapping the interacting domain between coronin 2B and UBF.
(**A**) Domain structure of coronin 2B and its truncated mutants. "UCC" represents the C-terminal regions containing the unique region and coiled-coil (CC) domain.
(**B**) FLAG-tagged coronin 2B and its truncated mutant (i.e., UCC and ΔUCC) were transfected into HEK293T cells, and cell lysates were incubated with UBF antibody for immunoprecipitation. Western blot analysis indicated UBF co-immunoprecipitated with full-length coronin 2B and the truncated mutant (i.e., ΔUCC) but not the truncated mutant alone (i.e., UCC).

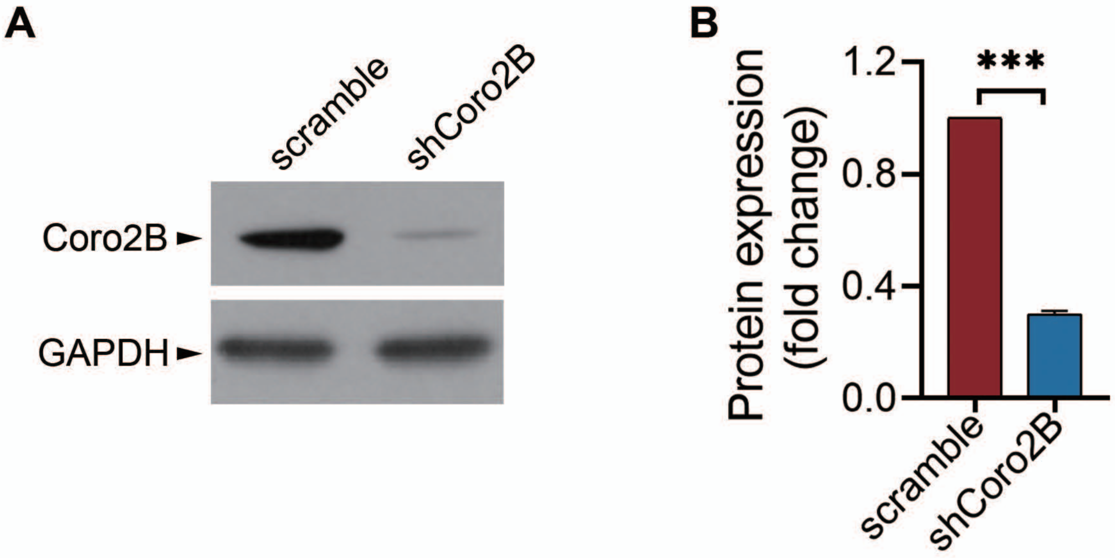

Supplementary Figure 3. Validation of the knockdown efficiency of shRNAs against coronin2B in cultural cortical neurons. Cortical neurons were infected with lentivirus expressing shCoro2B or corresponding scramble control. Western blot analysis indicates effective depletion of coronin 2B protein expression in neurons (**A**). The quantification is shown in (**B**). Data are mean ± SEM (from 3 independent experiments, Unpaired Student's t-test, ***$p < 0.001$)

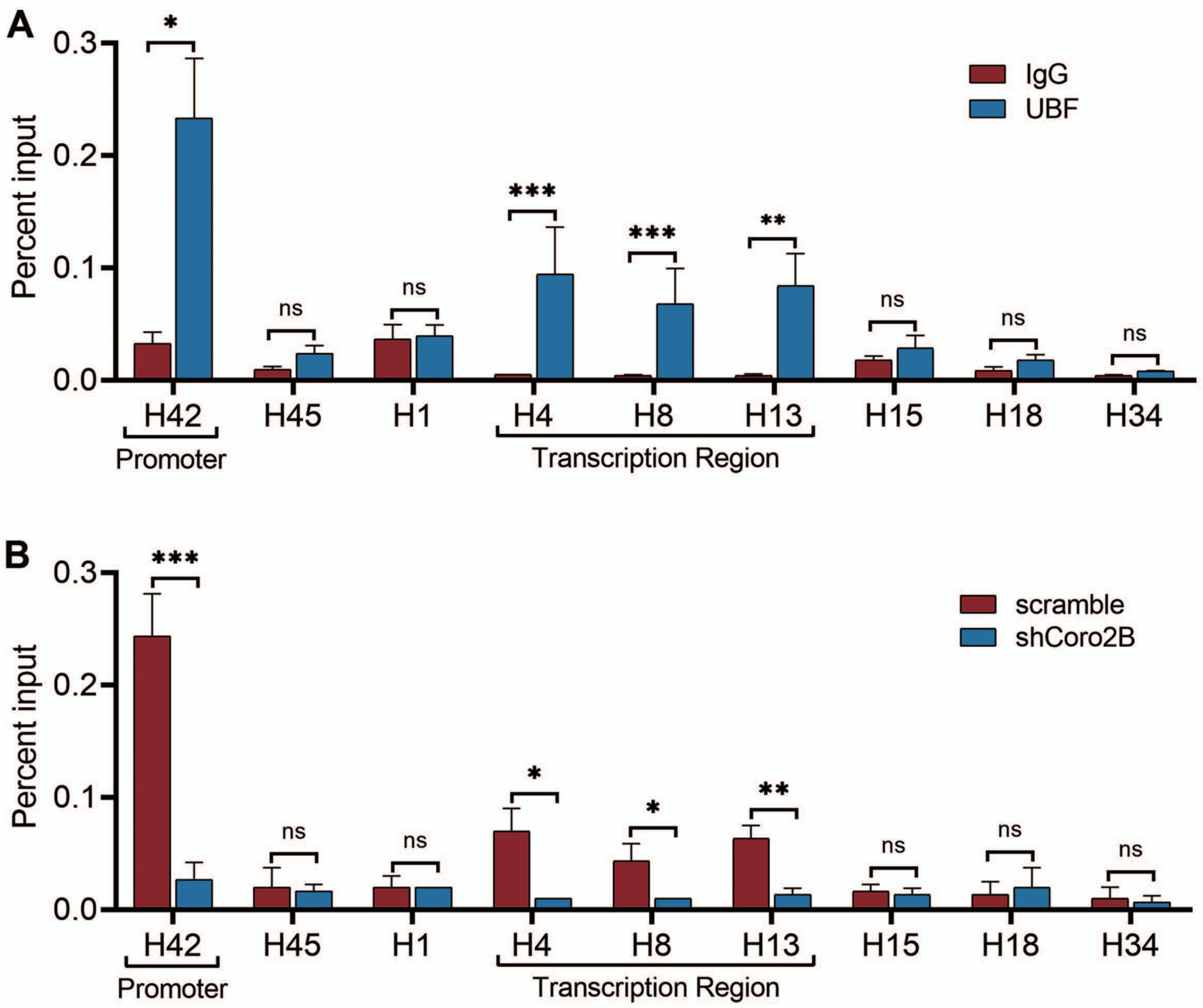

Supplementary Figure 4. The rDNA occupancy of UBF is compromised in coronin 2B-deficient neurons. Cortical neurons infected with shCoro2B lentivirus or corresponding scramble control were subjected to chromatin immunoprecipitation (ChIP) analysis. Then, rDNA was immunoprecipitated with antibodies against UBF and mouse IgG as a control. The precipitated DNA was assayed by RT-qPCR using primers spanning the entire rDNA repeat region. The results are presented as the percentage of input values normalized to the control. Data are mean ± SEM (from 3 independent experiments, Unpaired Student's t-test and Mann-Whitney U-test, *p < 0.05, **p < 0.01, ***p < 0.001, ns: not significant).

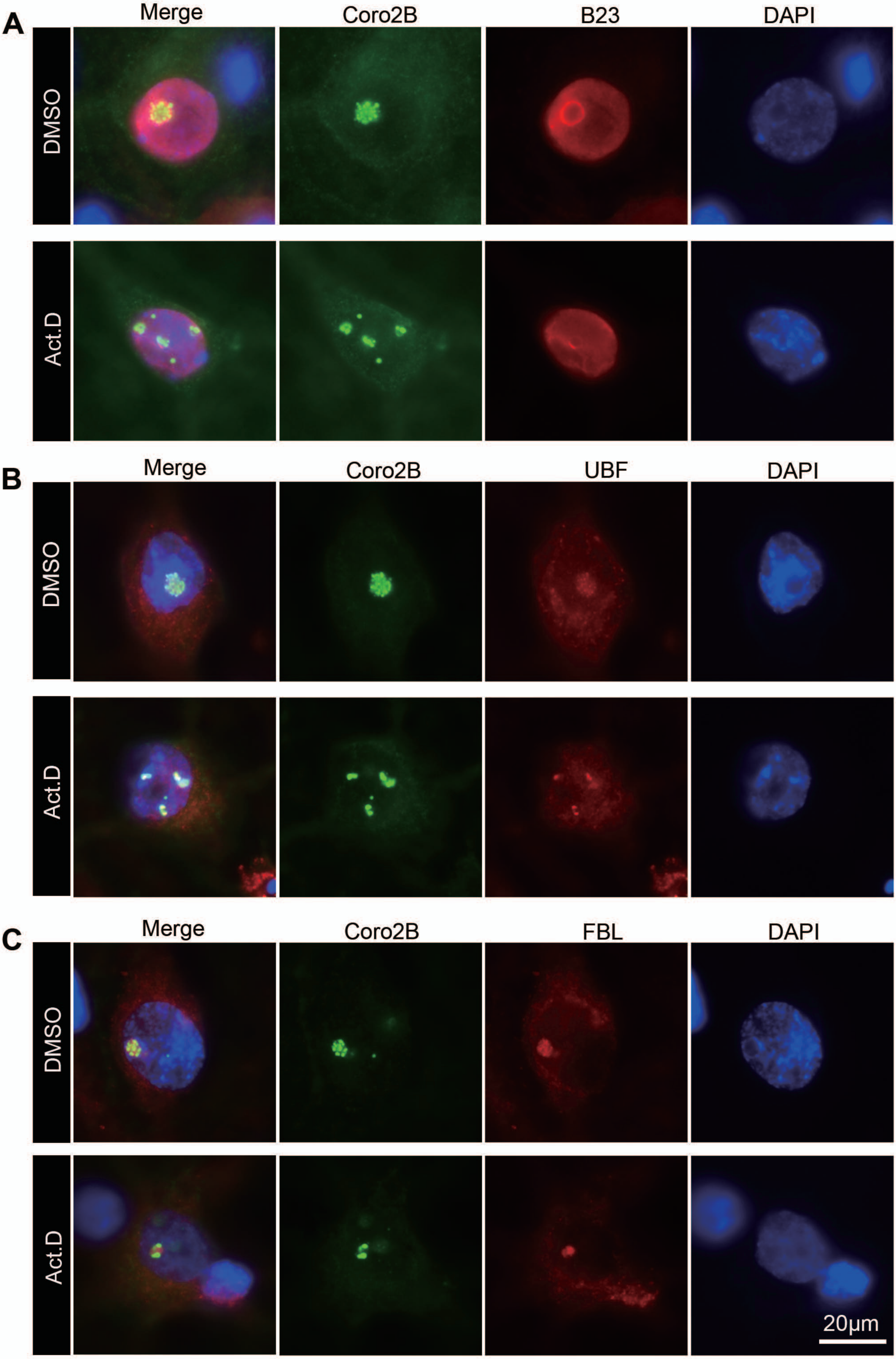

Supplementary Figure 5. Loss of nucleolar B23 in actinomycin D-treated neurons. Hippocampal neurons cultured for 14 days were treated with actinomycin D (Act. D, 8 nM) for 1 h. The localization of the nucleolar markers B23 (**A**), UBF (**B**), and FBL (**C**) were analyzed by immunofluorescence. Nucleoli were counterstained with DAPI. The reduction of nucleolar B23 staining indicates nucleolar stress. Scale bar: 20 μm.

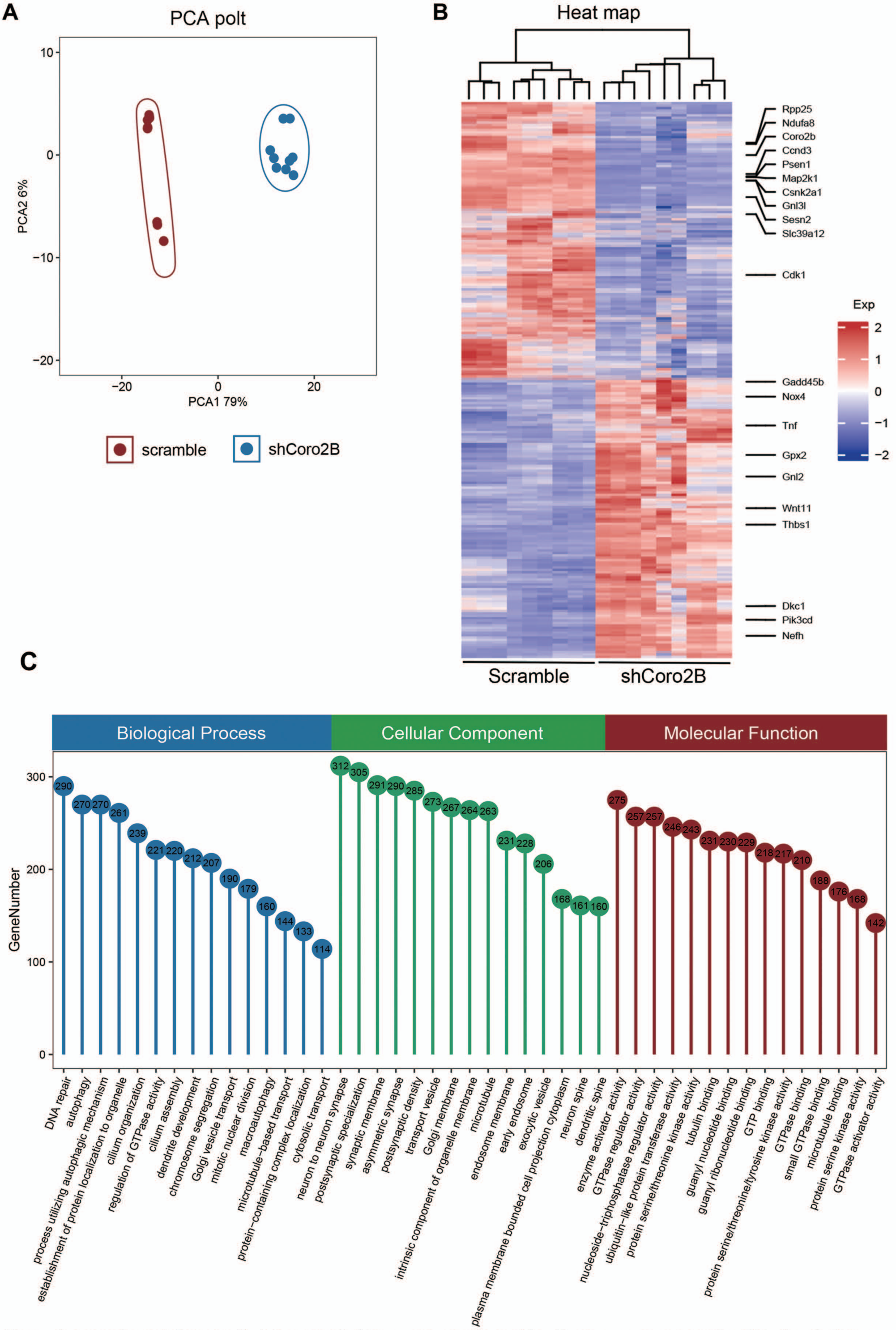

Supplementary Figure 6. Transcription program activation upon coronin 2B depletion.

(**A**) Principal component analysis of all transcripts was performed to visualize the data.

(**B**) A heatmap of differentially expression genes (cutoff: $p < 0.01$ and log2 [fold change] > 1 or < −1) is shown. Expression values are expressed as Z-score-transformed transcript counts.

(**C**).Differentially expressed genes assigned to enriched Gene Ontology (GO) terms (cutoff: $p < 0.01$)

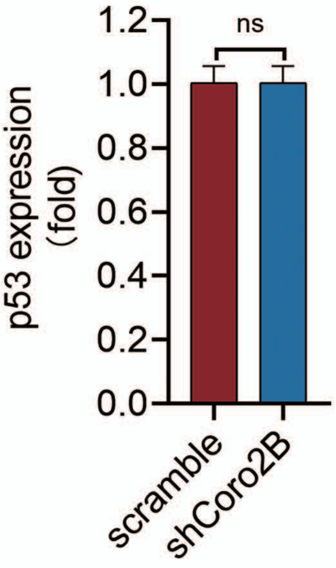

Supplementary Figure 7. The transcription level of p53 does not change upon coronin 2B knockdown. RNA was extracted from cortical neurons infected with shCoro2B lentivirus or scramble control. The transcription level of p53 mRNA was analyzed by qPCR. Data are mean ± SEM (from 3 independent experiments, Unpaired Student's t-test, ns: not significant).
