# Supplementary material for: Coronin 2B deficiency induces nucleolar stress and neuronal apoptosis
Source: Cell Death Dis. 2024 Jun 27;15(6):457. doi: 10.1038/s41419-024-06852-x (PMC11211331; doi:10.1038/s41419-024-06852-x)
Supplement: Supplementary file 3 — Supplementary Table 2 [file 41419_2024_6852_MOESM3_ESM.docx]

**Table S1. Primers used in this study**

| **rDNA transcription** | | |
| --- | --- | --- |
| 45S | Forward | GTTCCCGTGTTTTTCCGCTC |
|  | Reverse | AGTGCGTTCGAAGTGTCGAT |
| 28S | Forward | GTTCACCCACTAATAGGGAACGTGA |
|  | Reverse | GGATTCTGACTTAGAGGCGTTCAGT |
| 18S | Forward | GTTGGTTTTCGGAACTGAGGC |
|  | Reverse | GTCGGCATCGTTTATGGTCG |
| 5.8S | Forward | ACTCGGCTCGTGCGTC |
|  | Reverse | GCGACGCTCAGACAGG |
| **ChIP-PCR** | | |
| H1 | Forward | gaggcgcccaccccgcgacta |
|  | Reverse | AGGCCTCTCAAAGCTCCCCAC |
| H4 | Forward | CGACGACCCATTCGAACGTCT |
|  | Reverse | CTCCGGAATCGAACCCTGAT |
| H8 | Forward | AGTCGGGTTGCTTGGGAATGC |
|  | Reverse | CCCTTACGGTACTTGTTGACT |
| H13 | Forward | ACCTGGCGCTAAACCATTCGT |
|  | Reverse | AGAGACAAACCCTTGTGTCG |
| H15 | Forward | CGGGTGGTGGTAGCTGTAAT |
|  | Reverse | TTTTCGAGACAGGGTTCCTC |
| H18 | Forward | TCTTCCGAAGGTGCAGAGTT |
|  | Reverse | CTGCAGCTGTCCTCAGACAC |
| H34 | Forward | TGGCTAGTTTTCTGCCTGAA |
|  | Reverse | GTCAGTCAGTTGCCAGAGCCA |
| H42 | Forward | CGAGCACCAGAAAACAACAA |
|  | Reverse | CGAGCACCAGAAAACAACAA |
| H45 | Forward | CAGGTCGACCAGTTGTTCCT |
|  | Reverse | aggaaagtgacaggccacag |
| **Gene expression assay by qPCR assay** | | |
| Coronin 2B | Forward | AGCACGGAGAAGCCCTACCT |
|  | Reverse | GCAGACACATCCAACCCATG |
| Nefh | Forward | AACACCACTTAGATGGCGGG |
|  | Reverse | ACGTGGAGCGTTCAGCAATA |
| Wnt11 | Forward | CTCAAGACCCGCTACCTGTC |
|  | Reverse | TCTTGTTGCACTGCCTGTCT |
| Gpx2 | Forward | ACCAGTTCGGACATCAGGAGA |
|  | Reverse | ACAGGATGCTGATTCTGCCC |
| Ndufa8 | Forward | TCAGCTCAGCCGTGCTTAAA |
|  | Reverse | TACTCTGTGAAAGGCTCCGC |
| Map2k | Forward | GCTGAGTTGCAGGCTGTTTC |
|  | Reverse | CTCCAGGTTGGTCTCGGC |
| Tnf | Forward | ATGGGCTCCCTCTCATCAGT |
|  | Reverse | GCTTGGTGGTTTGCTACGAC |
| Nox4 | Forward | TGGCCAACGAAGGGGTTAAA |
|  | Reverse | ACACAATCCTAGGCCCAACA |
| Pik3cd | Forward | TGCTCCAAAGACATCCAGACT |
|  | Reverse | GCGCCTCTTACTGCCTGTTA |
| Slc39a12 | Forward | TCAGCTGCTCTTGTCAGGTG |
|  | Reverse | GCATGGAGCCCAAGGTTAGT |
| Psen1 | Forward | GAAGACCCGGAGGAAAGAGG |
|  | Reverse | GTTGTGTTCCAGTCCCCACT |
| Dkc1 | Forward | CGCGACTACGTGGACTACAG |
|  | Reverse | TGTCGCTATCGCGTTTCCTT |
| Gnl2 | Forward | CCACCAAAAGGGCAAGGAAG |
|  | Reverse | TAGTAGCGCACACCGACTTT |
| Rpp25 | Forward | GCCTCGCCATCCTACTTTCA |
|  | Reverse | CTCCTCTTGGACGTCGACAC |
| Gnl3l | Forward | GCTTCTATACGCCACCACCG |
|  | Reverse | TCCCACGGCTAAGCATTCC |
| Csnk2a1 | Forward | CGATACGACCACCAGTCTCG |
|  | Reverse | TGGCACTGAAGAAATCCCTGA |
| Thbs1 | Forward | TACAGATGGCGTCTCAGCCA |
|  | Reverse | GGTGATTAGGAGTCTCGGCAC |
| Gadd45b | Forward | CCTCCTGGTCACGAACTGTC |
|  | Reverse | GGACCCACTGGTTATTGCCT |
| Ccnd3 | Forward | GACTCGCTAGCCCGCAC |
|  | Reverse | GCACGCACTGGAAGTAGGAG |
| Cdk1 | Forward | GGAACAGAGAGGGTCCGTTG |
|  | Reverse | GAGATTTCCCGGATTGCCGT |
| Sesn2 | Forward | AGGAATGGCACCTGGAACAG |
|  | Reverse | ATACGCATGCTCATCCCTGG |
