# Supplementary material for: Coronin 2B deficiency induces nucleolar stress and neuronal apoptosis
Source: Cell Death Dis. 2024 Jun 27;15(6):457. doi: 10.1038/s41419-024-06852-x (PMC11211331; doi:10.1038/s41419-024-06852-x)

Relate to Figure 1A

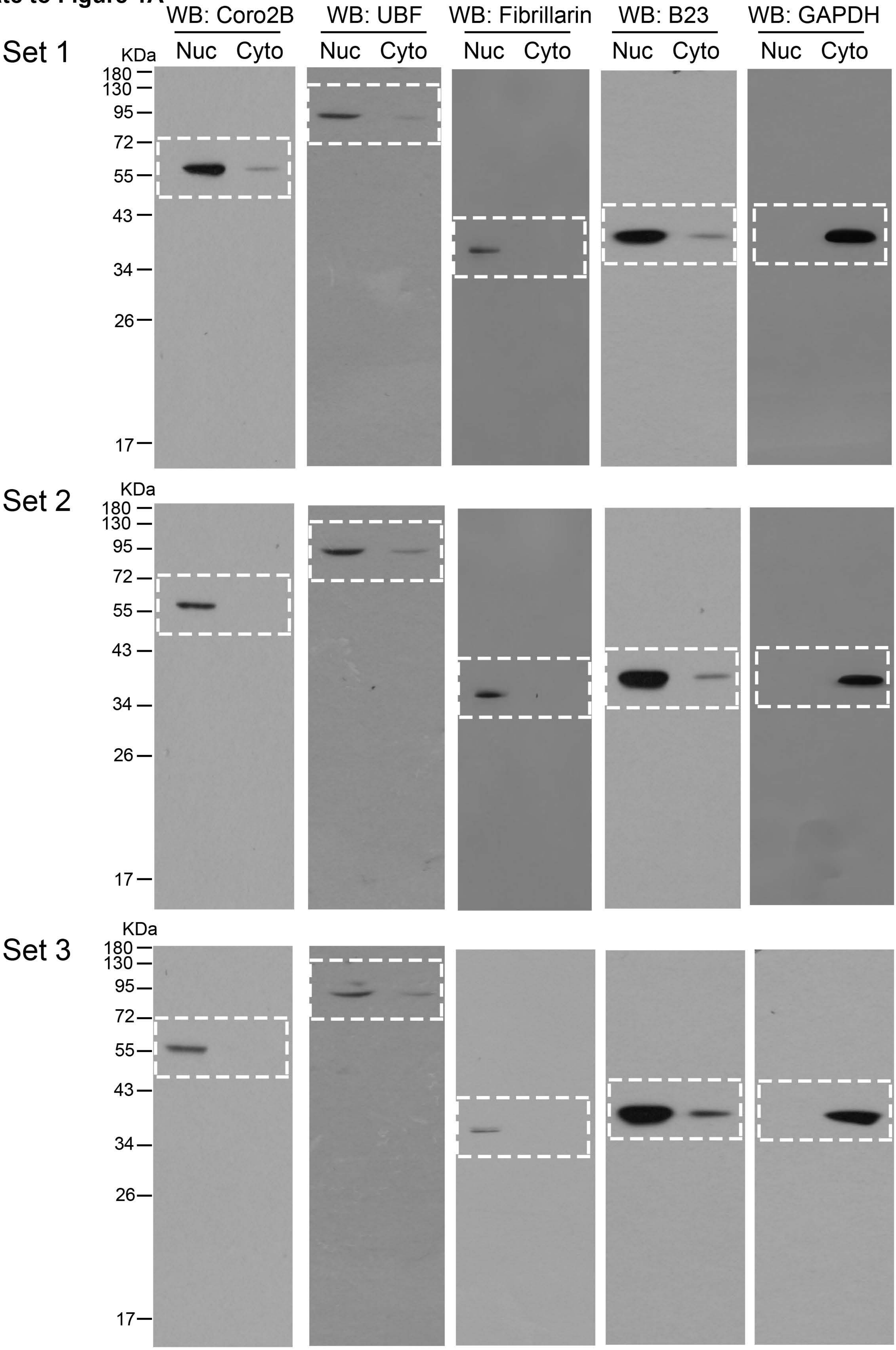

Relate to Figure 1C

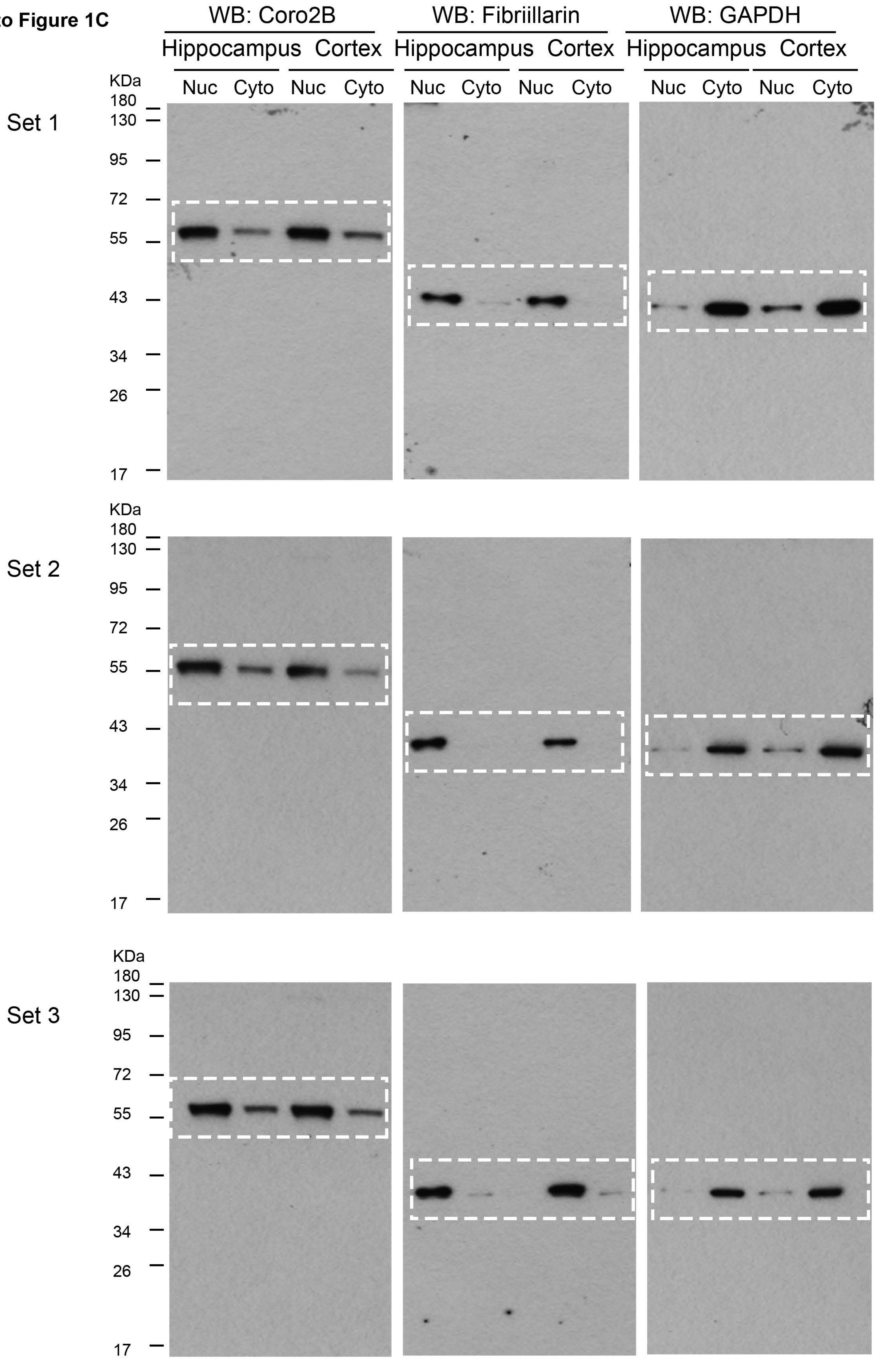

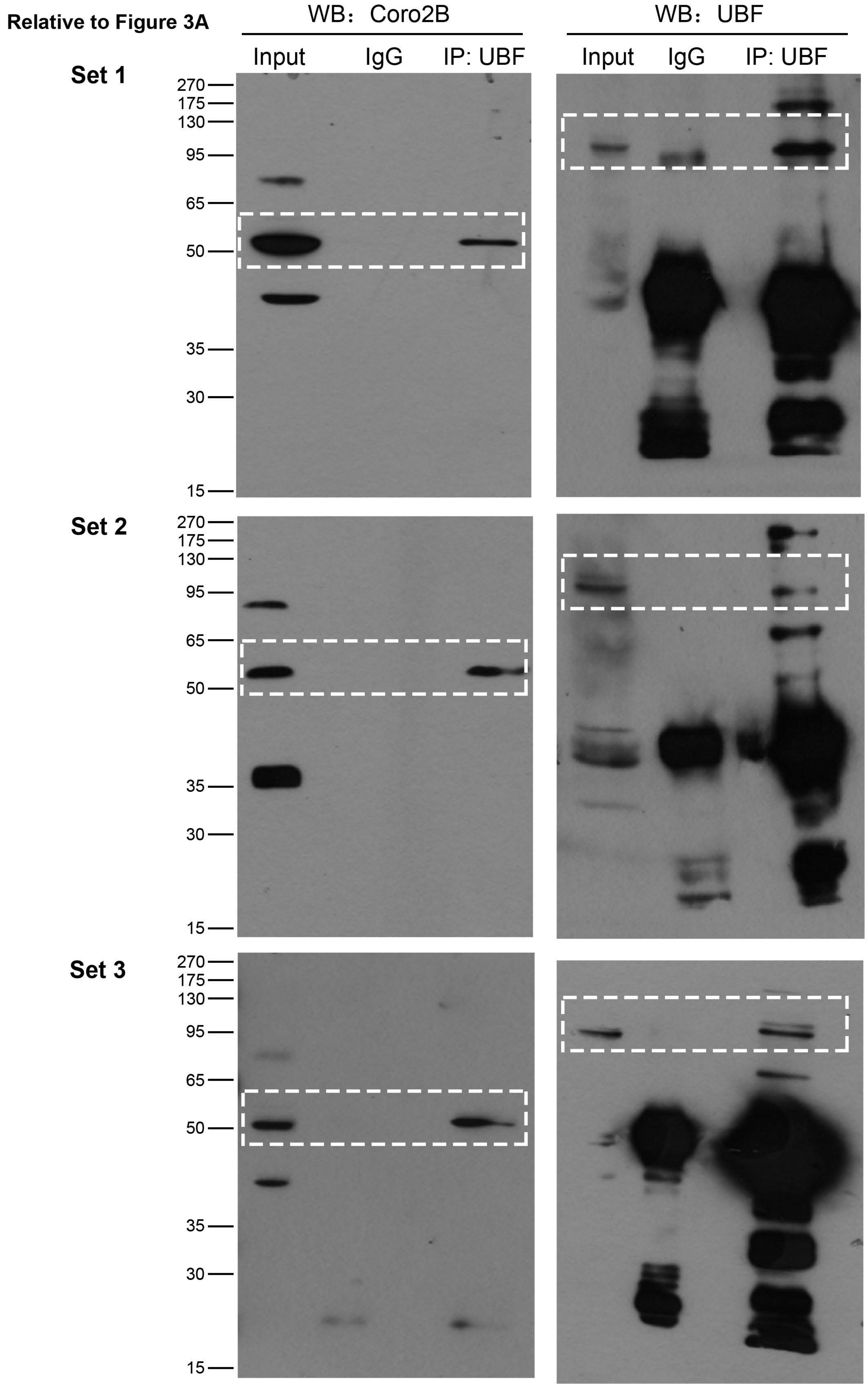
Relative to Figure 3A
WB：Coro2B
WB：UBF
Input
IgG
IP: UBF
Input
IgG
IP: UBF
Set 1
Set 2
Set 3
270
175
130
95
65
50
35
30
15

Relate to Figure 3B

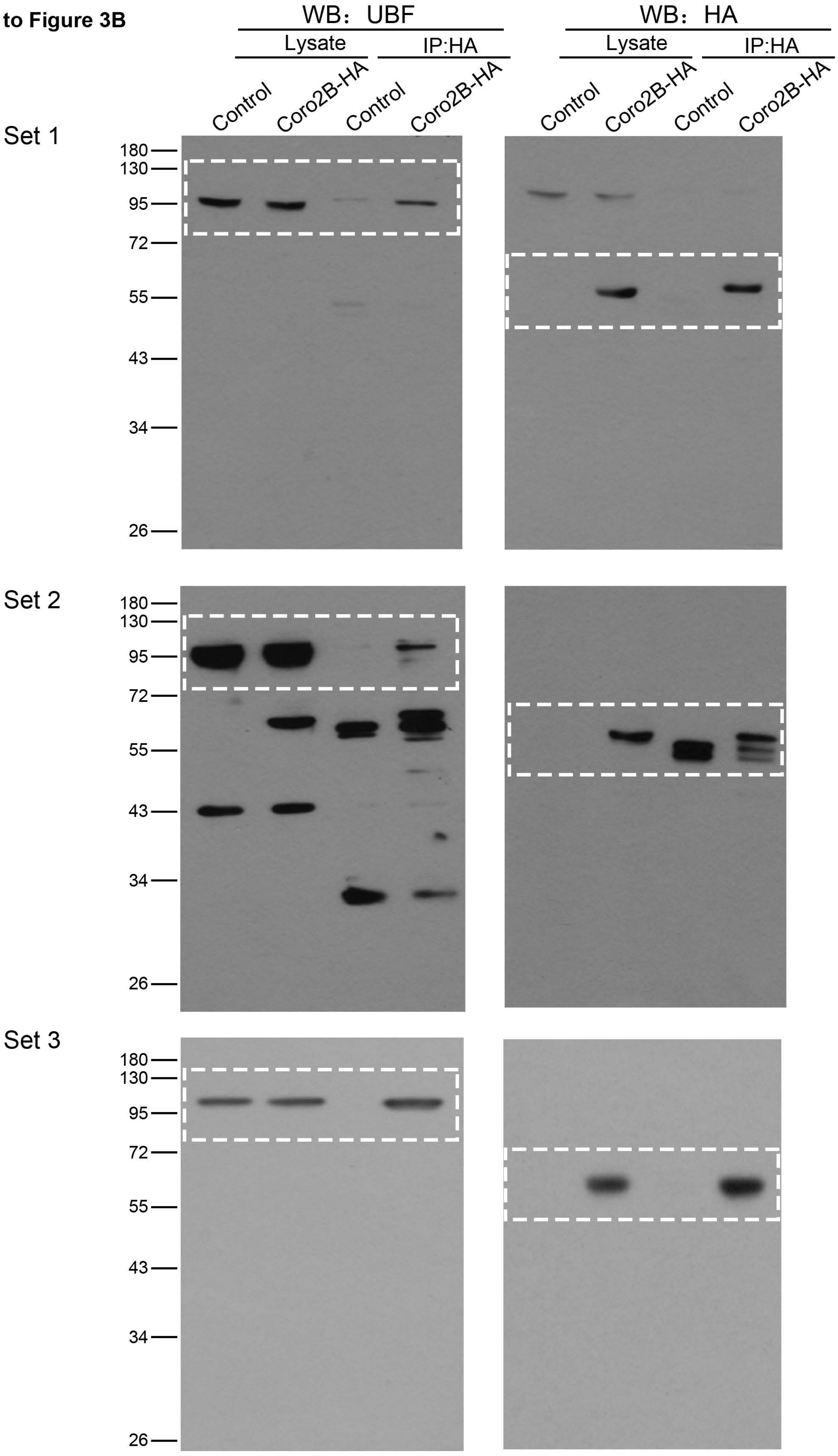

Relate to Figure 3C

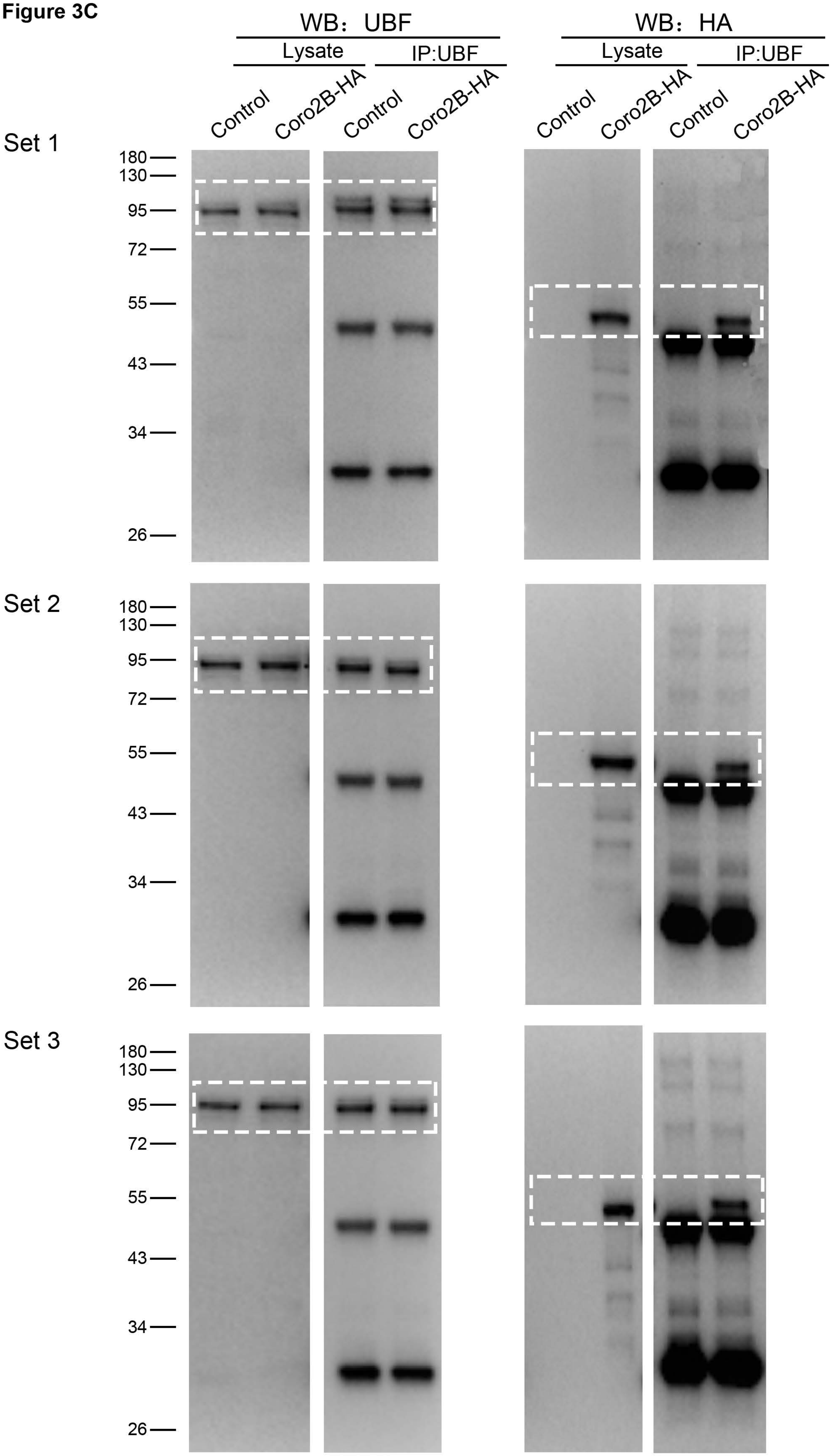

Relate to Figure 3D and 3F

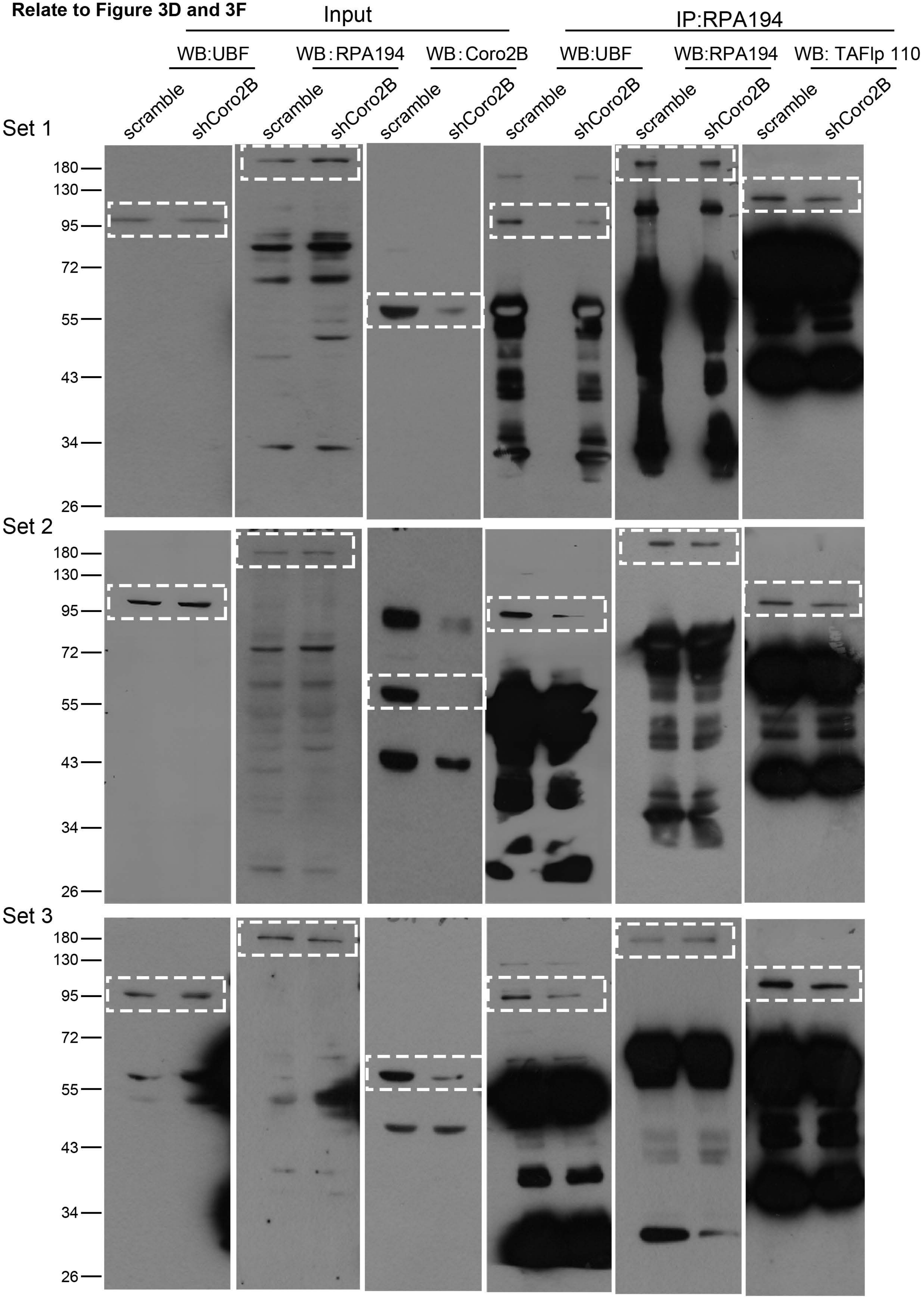

Relate to Figure 4D

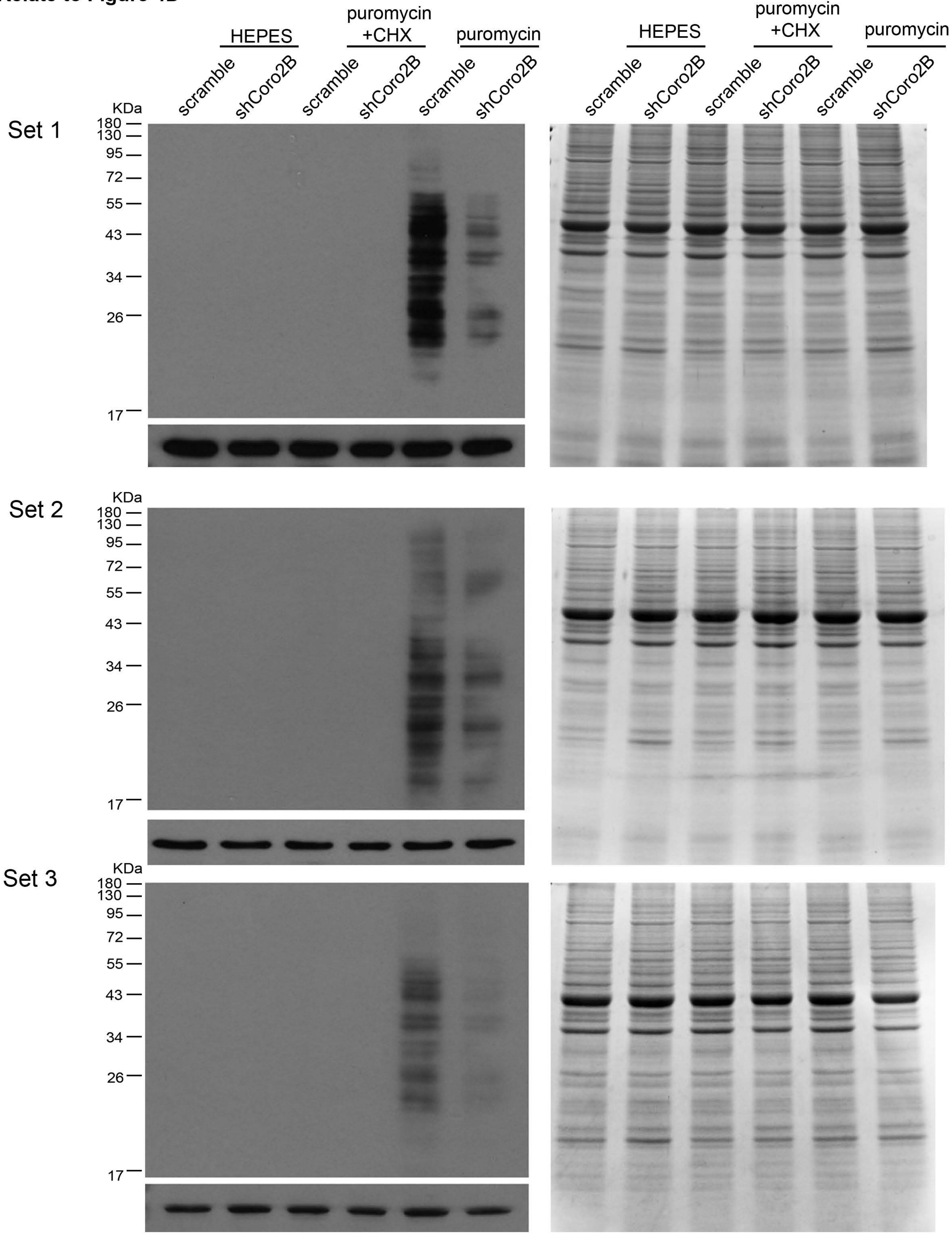

Relate to Figure 6A

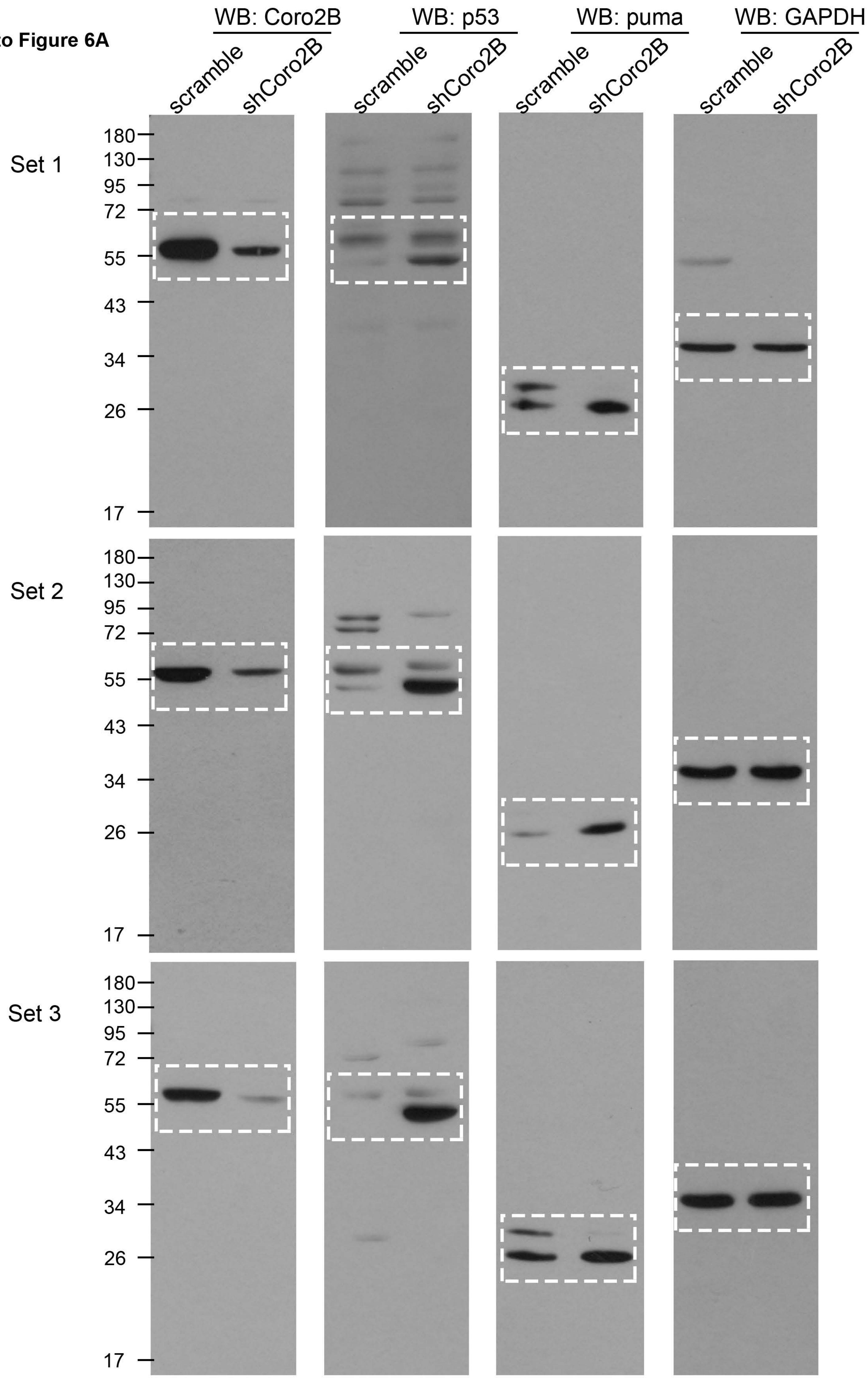

Relate to Figure 6C

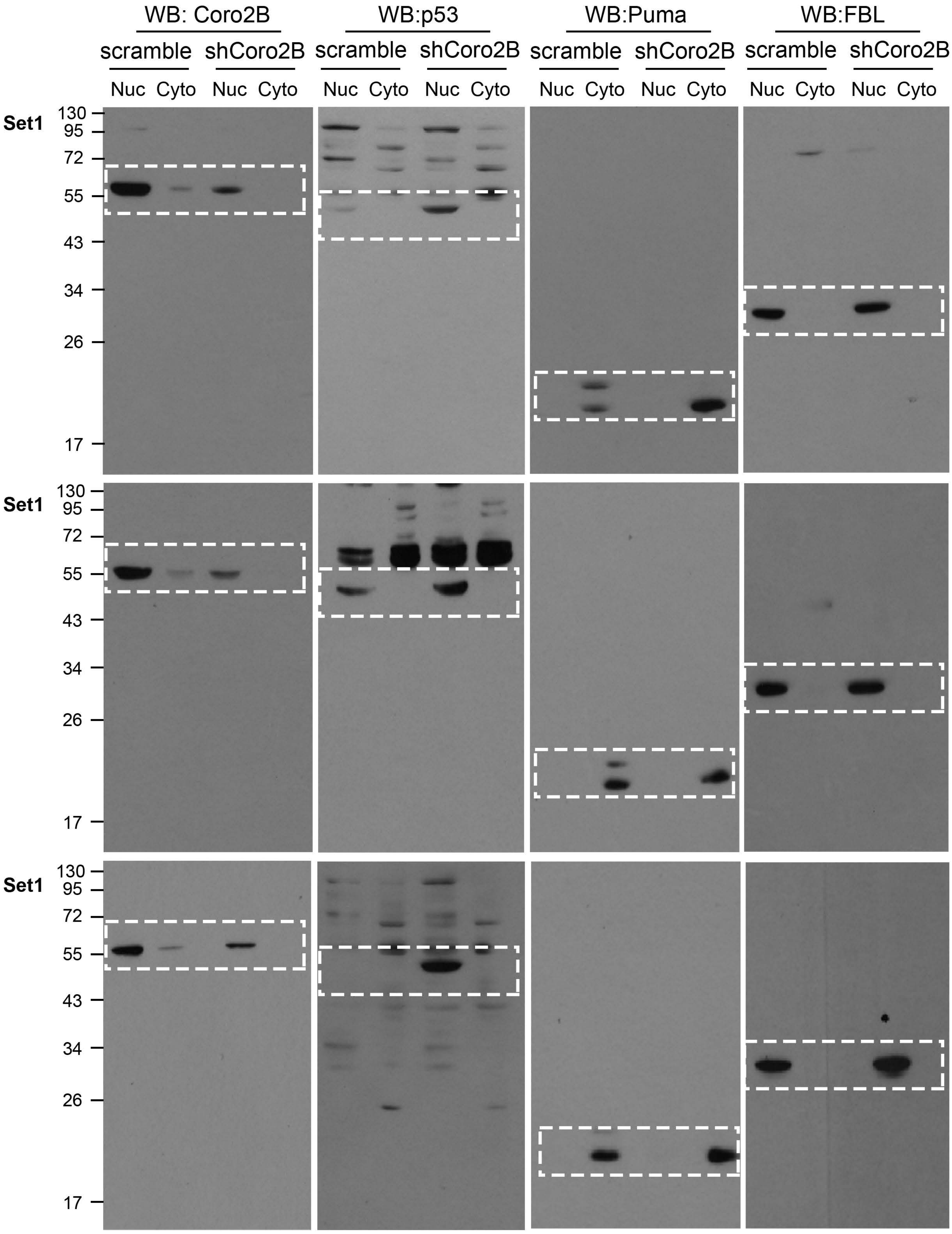

Relate to Figure 6E

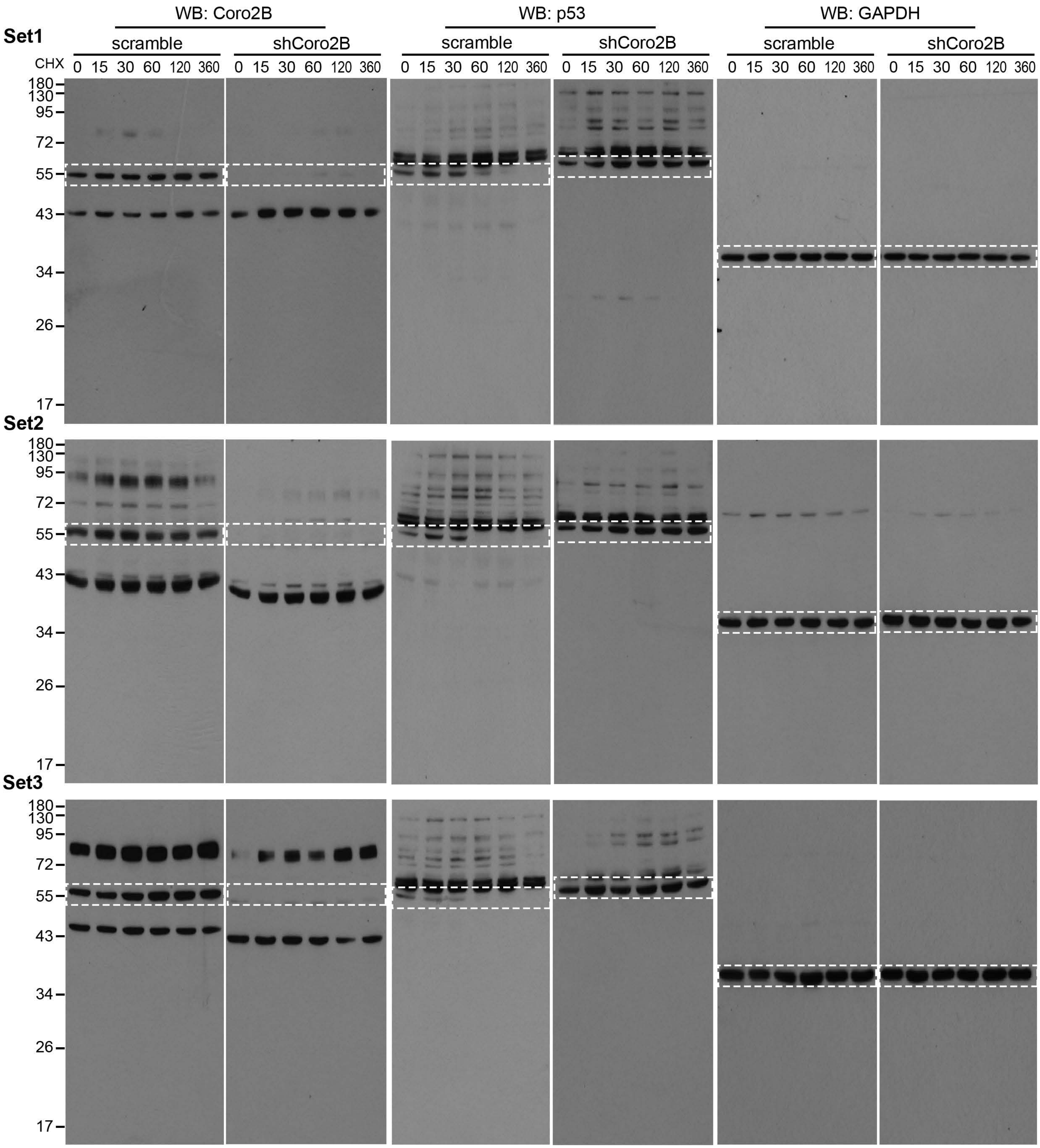

Relate to Figure 6G

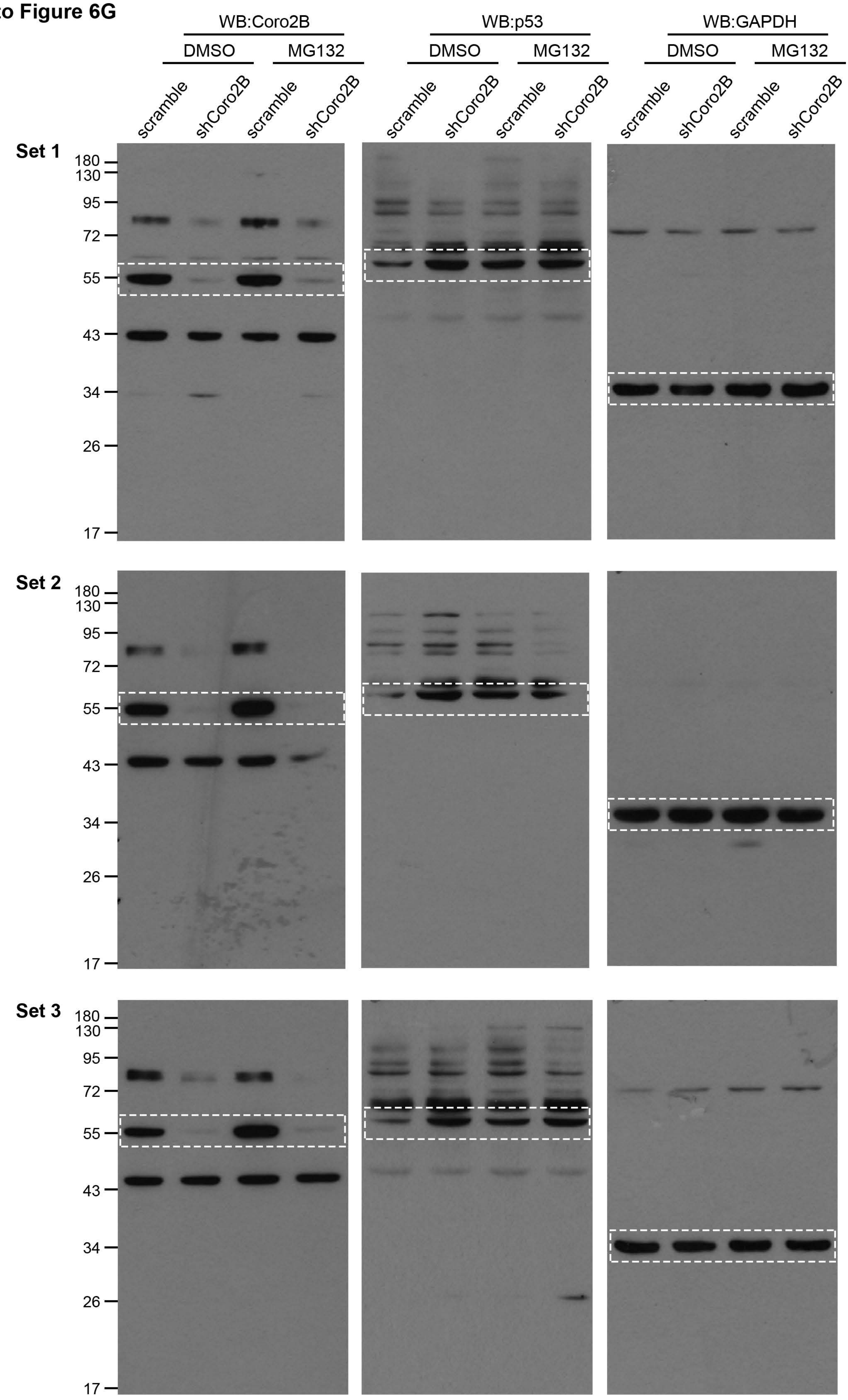

Relate to Figure 7A

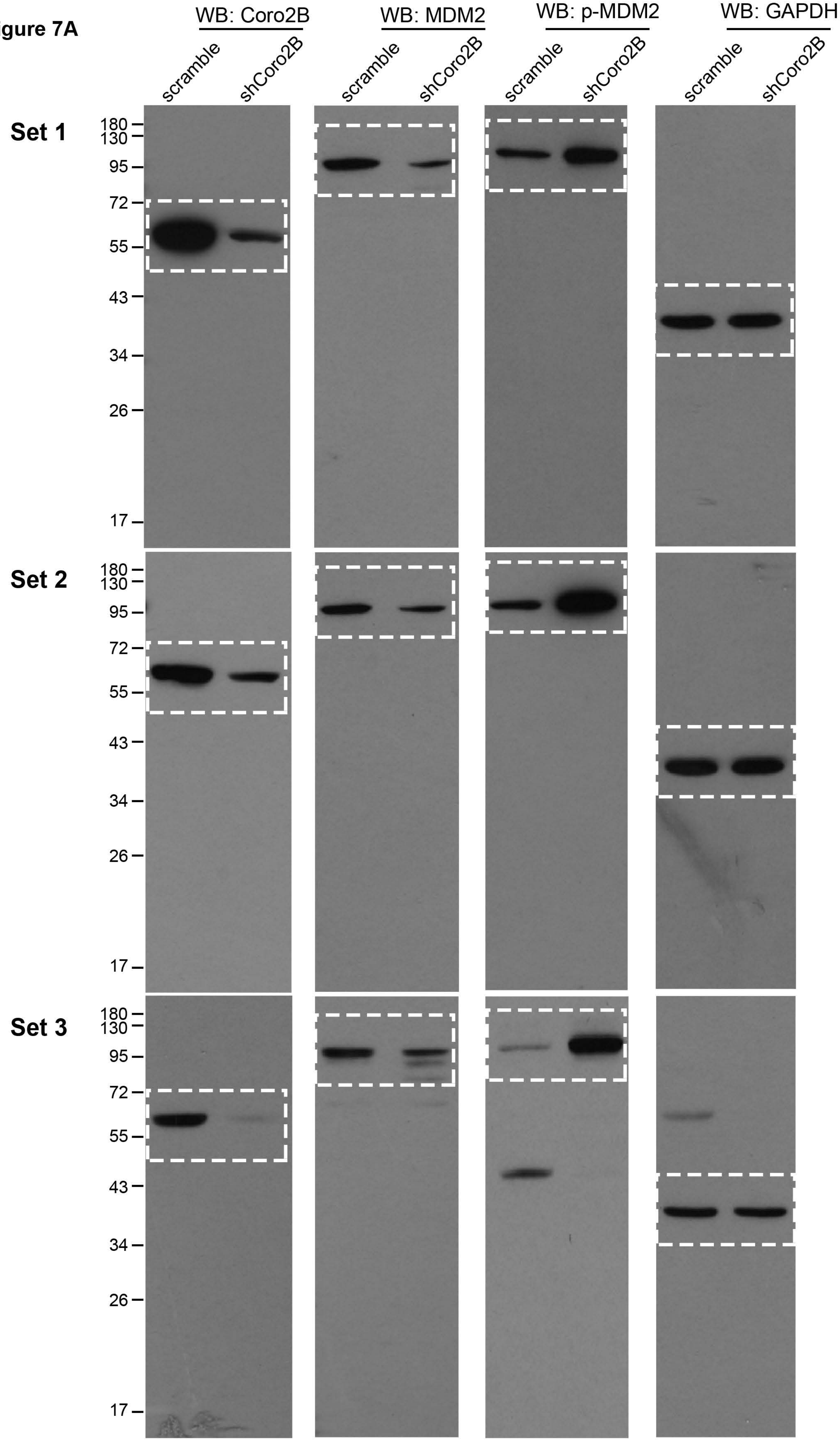

Relate to Figure 7C

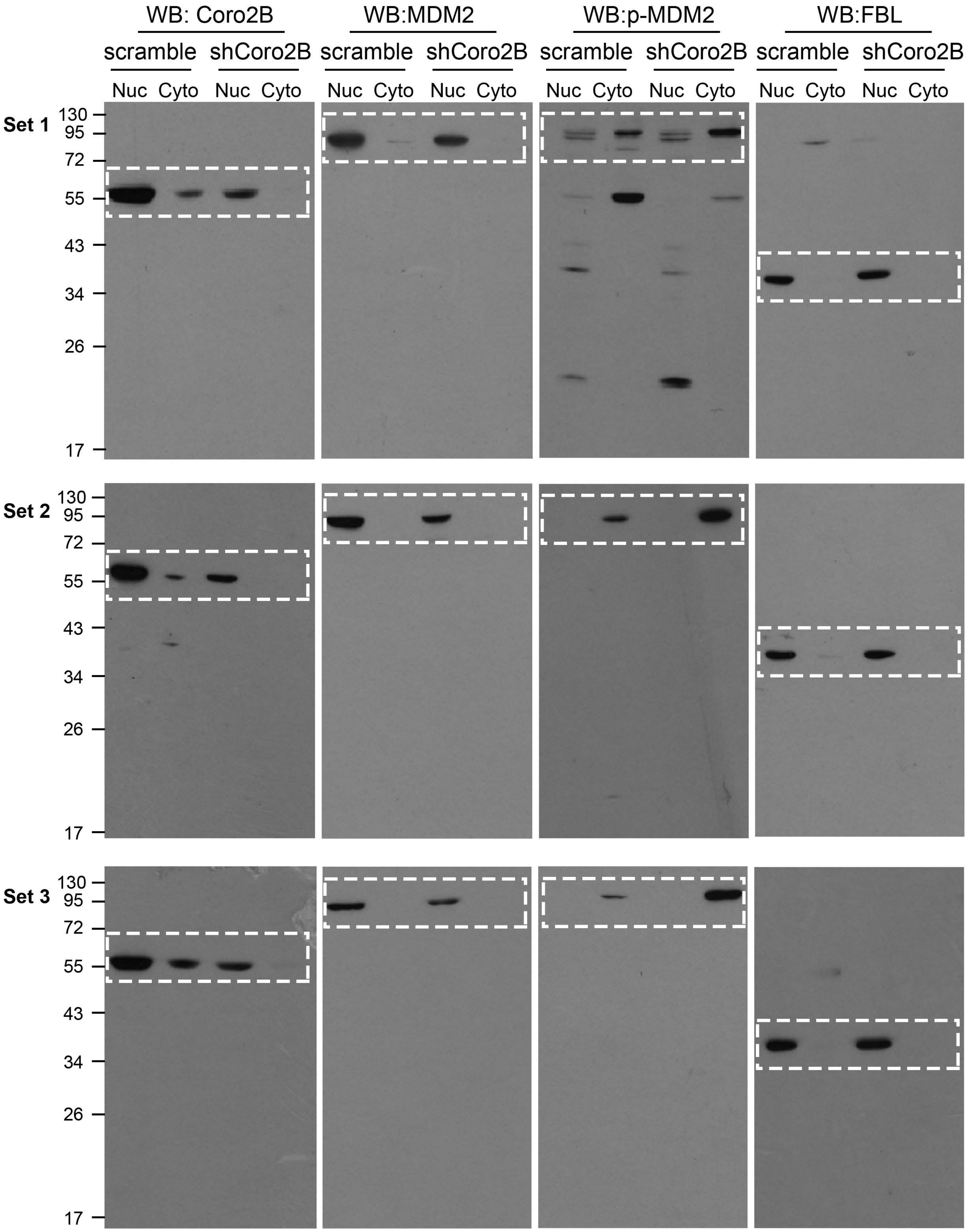

Relate to Figure 8F

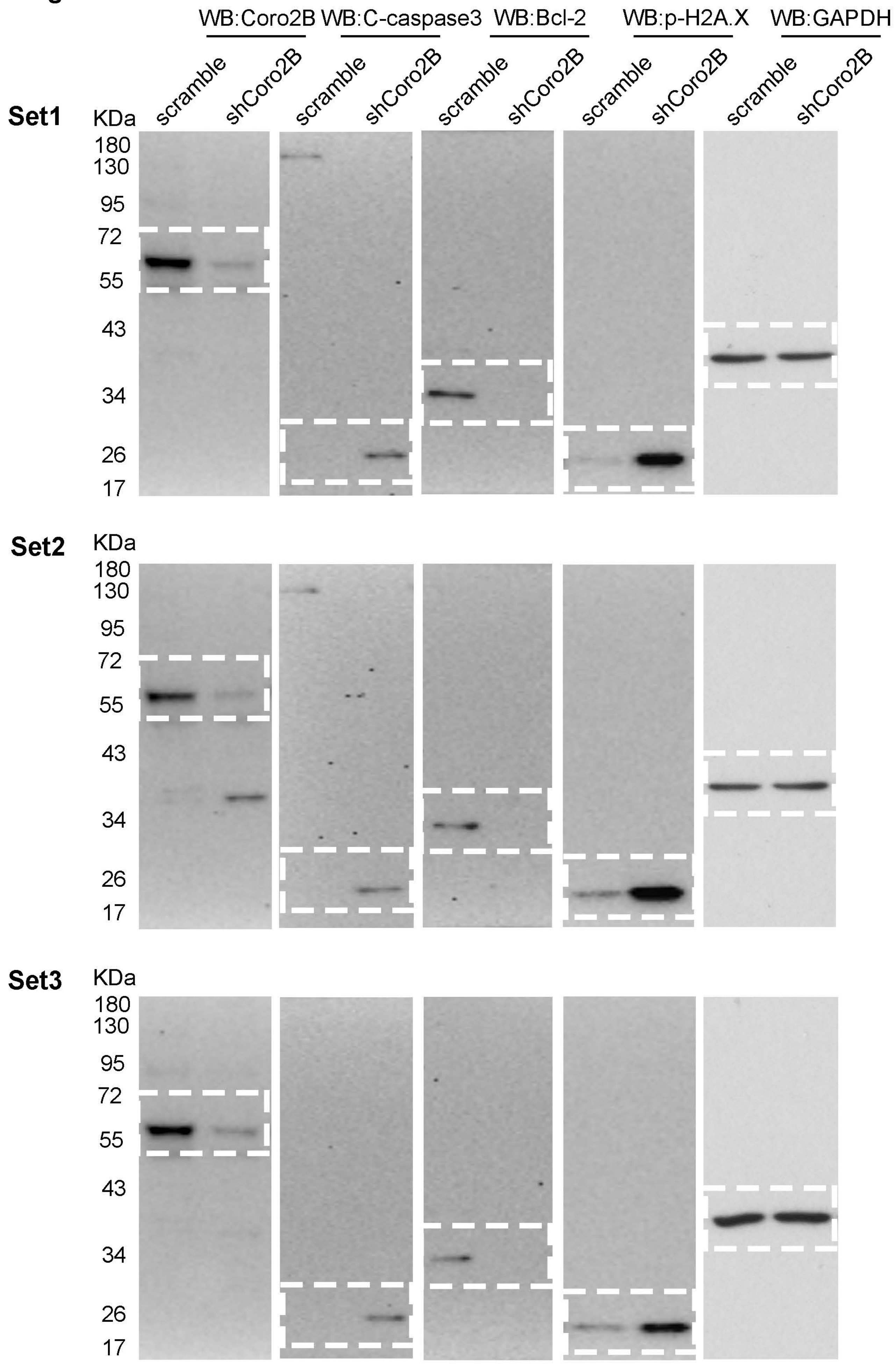

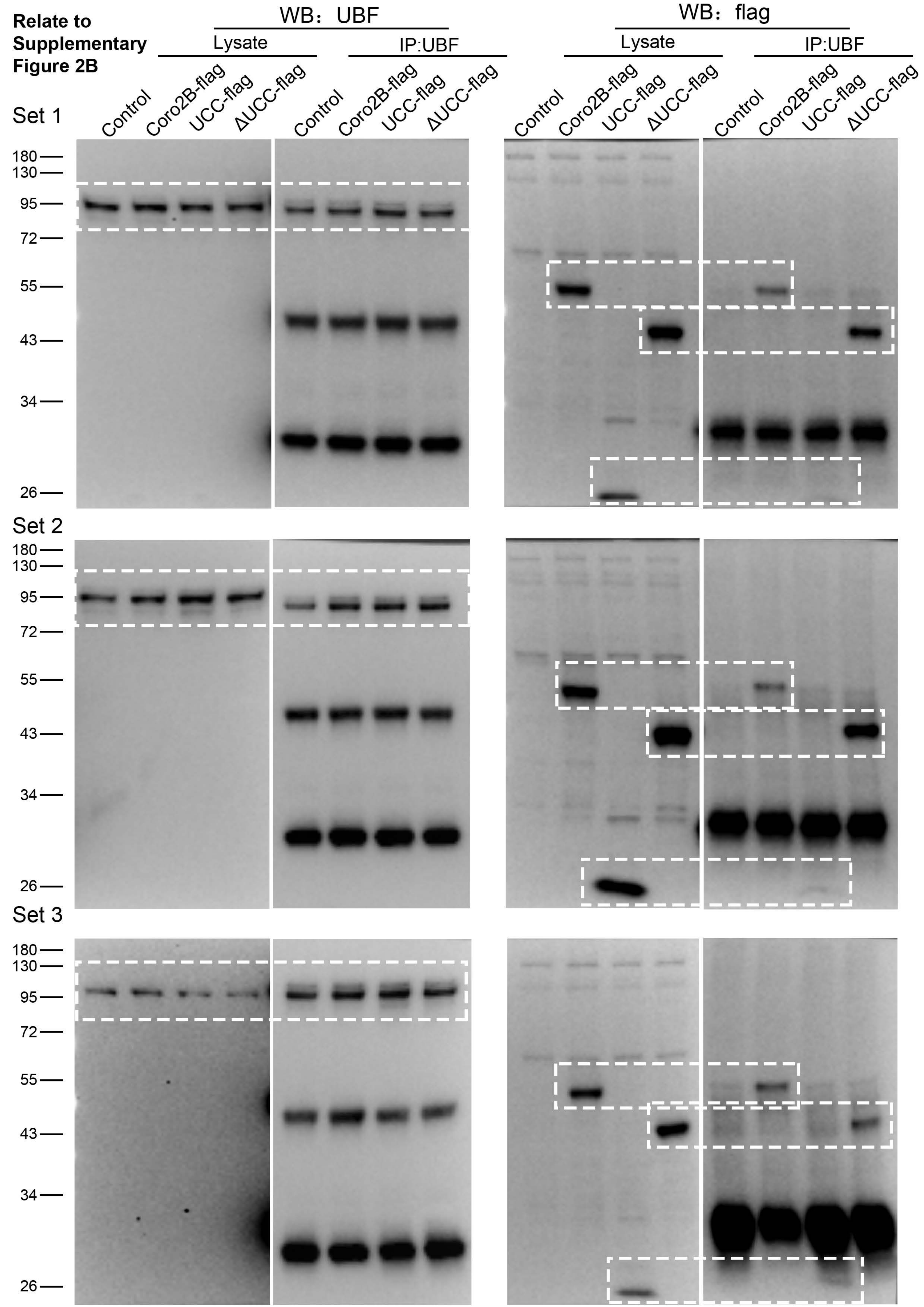
Relate to Supplementary Figure 2B
WB：UBF
WB：flag
Lysate
IP:UBF
Control
Coro2B-flag
UCC-flag
ΔUCC-flag
Set 1
Set 2
Set 3
180
130
95
72
55
43
34
26

Relate to Supplementary Figure 3A

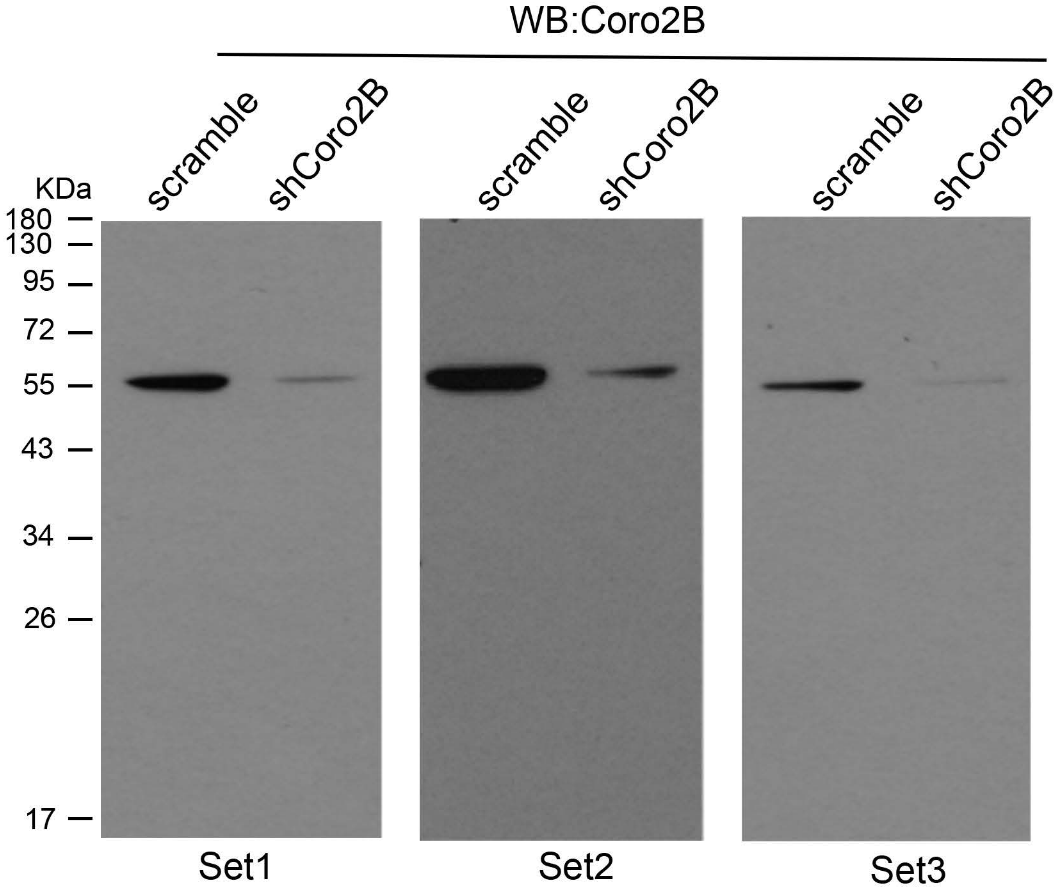

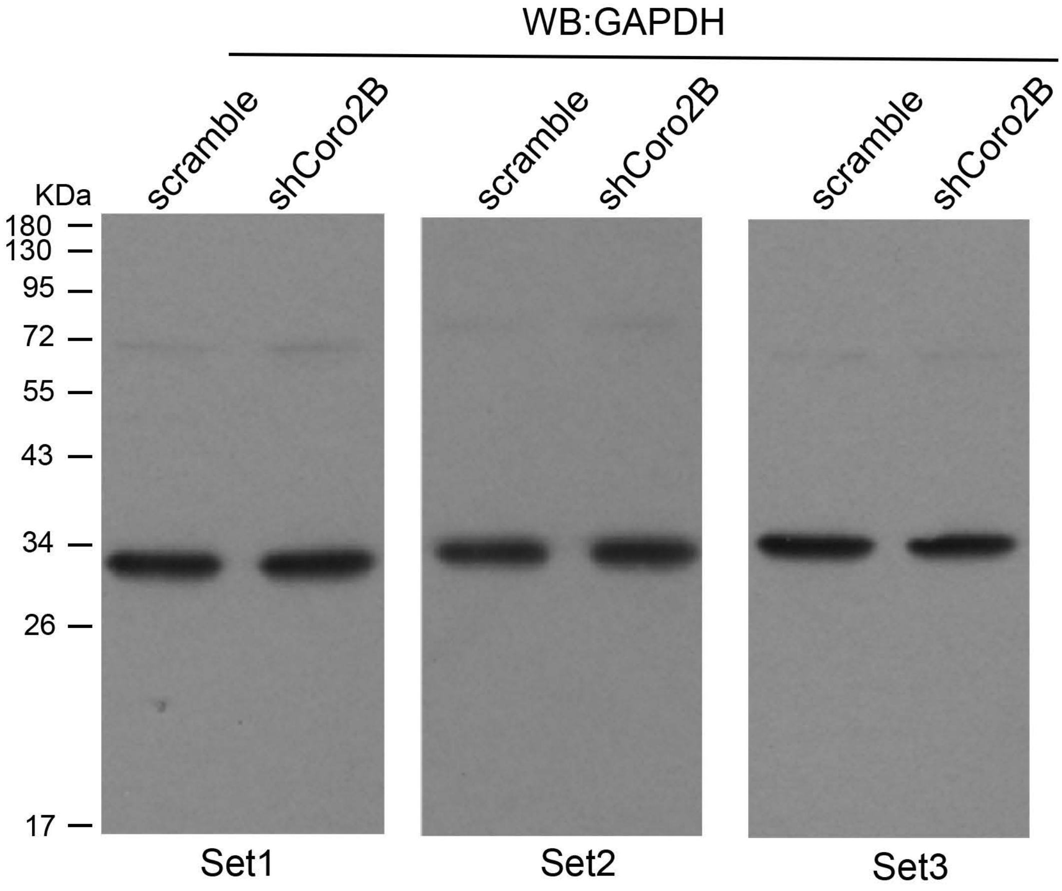

Supplement: Supplementary file 5 — Uncropped Western blots [file 41419_2024_6852_MOESM5_ESM.pdf]
